# Short and long-term effects of robot-assisted therapy on upper limb motor function and activity of daily living in patients post-stroke: a meta-analysis of randomized controlled trials

**DOI:** 10.1186/s12984-022-01058-8

**Published:** 2022-07-21

**Authors:** Liping Zhang, Gongwei Jia, Jingxi Ma, Sanrong Wang, Li Cheng

**Affiliations:** 1 Department of Rehabilitation, The Second Affiliated Hospital of Chongqing Medical University, 76 Linjiang Road, Yuzhong, Chongqing, 400010 China; 2 Department of Neurology, Chongqing General Hospital, University of Chinese Academy of Sciences, Chongqing, 400013 China; 3 Chongqing Key Laboratory of Neurodegenerative Diseases, Chongqing, 400013 China; 4 Department of Health Management, The Second Affiliated Hospital of Chongqing Medical University, 76 Linjiang Road, Yuzhong, Chongqing, 400010 China

**Keywords:** Stroke, Rehabilitation, Robot-assisted therapy, Upper limb, Meta-analysis

## Abstract

**Objective:**

To investigate the effect of robot-assisted therapy (RAT) on upper limb motor control and activity function in poststroke patients compared with that of non-robotic therapy.

**Methods:**

We searched PubMed, EMBASE, Cochrane Library, Google Scholar and Scopus. Randomized controlled trials published from 2010 to nowadays comparing the effect of RAT and control treatment on upper limb function of poststroke patients aged 18 or older were included. Researchers extracted all relevant data from the included studies, assessed the heterogeneity with inconsistency statistics (I^2^ statistics), evaluated the risk of bias of individual studies and performed data analysis.

**Result:**

Forty-six studies were included. Meta-analysis showed that the outcome of the Fugl-Meyer Upper Extremity assessment (FM-UE) (SMD = 0.20, P = 0.001) and activity function post intervention was significantly higher (SMD = 0.32, P < 0.001) in the RAT group than in the control group. Differences in outcomes of the FM-UE and activity function between the RAT group and control group were observed at the end of treatment and were not found at the follow-up. Additionally, the outcomes of the FM-UE (SMD = 0.15, P = 0.005) and activity function (SMD = 0.32, P = 0.002) were significantly different between the RAT and control groups only with a total training time of more than 15 h. Moreover, the differences in outcomes of FM-UE and activity post intervention were not significant when the arm robots were applied to patients with severe impairments (FM-UE: SMD = 0.14, P = 0.08; activity: SMD = 0.21, P = 0.06) or when patients were provided with patient-passive training (FM-UE: SMD = − 0.09, P = 0.85; activity: SMD = 0.70, P = 0.16).

**Conclusion:**

RAT has the significant immediate benefits for motor control and activity function of hemiparetic upper limb in patients after stroke compared with controls, but there is no evidence to support its long-term additional benefits. The superiority of RAT in improving motor control and activity function is limited by the amount of training time and the patients' active participation.

**Supplementary Information:**

The online version contains supplementary material available at 10.1186/s12984-022-01058-8.

## Introduction

Stroke is the main cause of mortality and disability worldwide [1]. Even though the mortality rate significantly decreased from 1990 to 2019 [2], a growing number of survivors are living with motor function loss and require nursing care [1]. Impairment of upper limb function is a common problem among post-stroke patients [3]. According to the International Classification of Functioning, Disability, and Health (ICF), upper limb function can be divided into body function and structures, activity (capacity and performance), and participation [4]. The impairment of motor function could limit activity and result in difficulty in reintegrating into society for poststroke patients [5]. Several approaches for the recovery of motor function exist, but the debate about the effect of these treatments is ongoing [6]. Traditional neurological treatments, such as Bobath, proprioceptive neuromuscular facilitation (PNF) therapy, and other upper limb exercises, are well known and are common treatments for rehabilitation. However, comparing with these traditional rehabilitation treatments, robotic devices may be advantageous in terming of the output of objective measures such as speed, torque, range of motion, position, and others to evaluate and monitor the patient's improvement, and the customization of treatment sessions regarding different levels of movement impairment of patients [7]. In addition, the advantage of these manual therapies most depends on the clinical skill of therapist and hardly be reproducible, whereas RAT has high-consistency and reproducibility to allow its widespread use[8]. Moreover, there is strong evidence supporting that intensive, highly repetitive, task-oriented training promotes motor function recovery after stroke [6]. The intensity and repetition of traditional rehabilitation programs carried out by physical and occupational therapists cannot reach such a level [9]; hence, assistance from rehabilitation tools is needed. Arm robots with specialized technological machines can effectively provide high-intensity, highly repetitive_,_ functional, and precise exercises to better improve motor control function, strength, and accuracy of movement compared with traditional manual neurological treatments [9].

Although a better therapeutic effect of robot-assisted therapy (RAT) on motor and activity function has been reported [7,10–13], disparate effects and heterogeneities between trials were found depending on the phase of poststroke [14], the amount of training [15], the control system of the robots (e.g., patient-passive control robots versus patient-active control robots) [16] and the targeted joints of robots (e.g., proximal upper limb versus distal approach) [17], several meta-analyses have discussed the influence of stage of stroke [18–22] and the targeted joints of robots [20,22,23] on benefits of RAT on motor control and activity function, but few study focused on the level of impairment of patients, and the parameters of RAT such as amount of training time and the control system of the robots, thus we performed comprehensive analysis to discuss those factors to try to determine the optimal treatment parameters.

It is known that the control systems of arm robots can influence the therapeutic effect [16], the arm robots can be divided into patient-passive control robots and patient-active control robots according to the control strategies of robots. Patient-passive control robots mainly deliver automated practical movements to patients, and patient-active control robots can monitor and evaluate the physical parameters and performance of voluntary motion of patients [24] and then provide assistance as needed to complete the movement initiated by patients [25]. In the latter strategy, patients pay more attention to and put more effort into the training and more actively participate in the practice [26], which is essential for improving cortical activity, excitability and motor performance ^[[[[[27]]]]]^. Active participation is influenced by the level of impairment, the mechanical properties of the robot, the control strategies, the training mode of the robot, the instructions of the therapist and various other factors, therefore, we conducted a subgroup analysis to investigate the effect of training mode and impairment level on the superiority of RAT.

Moreover, most clinical trials have focused on the outcomes post intervention, and few studies discussed the long-term effect of RAT on activity function at follow-up. However, the changes in motor and activity function were different at the end of treatment and at follow-up [28,29], and a previous study [30] found that the gains in the Fugl-Meyer Upper Extremity (FM-UE) and Functional Independence Measure (FIM) between the robotic group and the control group were significantly different at discharge but not at the six-month follow-up.

Therefore, we performed this systematic review to investigate the effect of RAT on motor control and activity and to further discuss whether the effect of RAT persists longer than the three-month follow-up and how the amount of training, level of impairment and training mode influence the effect, this research might provide evidence for therapist to determine the optimal parameter such as total training time and training mode for clinical application of RAT.

## Methods

This systematic review was conducted according to the Preferred Reporting Items for Systematic Reviews and Meta-Analyses (PRISMA) guidelines. We have registered this review in PROSPERO (registered ID CRD42021189643).

Search Strategy and Selection of Studies:

We searched the literature in five databases (PubMed, Cochrane Library, EMBASE, Google Scholar and Scopus) for randomized controlled trials (RCTs) published from 2010 to nowadays. Our research is based on the following overarching participant, intervention, comparison and outcome (PICO) format:

Does robot-assisted therapy (RAT) (intervention) better improve upper limb motor control or activity (outcome) than non-robotic therapy (comparison) in adult poststroke patients (participant) after treatment or during the follow-up period (≥ three months)?

The search terms we used were “robot-assisted therapy" (robotic therapy (RT), exoskeleton, robot-supported, rehabilitation robot, robotic rehabilitation, robotic device, robot-aided rehabilitation), "upper limb" (upper extremity, arm, arm injuries, hand, hand injuries, shoulder, shoulder injuries, elbow, axilla elbow, forearm injuries, forearm, finger, finger injuries, wrist injuries, wrist), and "stroke" (middle cerebral artery infarction, intracranial hemorrhage, hemiplegia, cerebral vascular accident (CVA), cerebral vascular disorders, paresis).

### Inclusion criteria

Two researchers independently evaluated the studies, and studies were included if they met the following criteria: (1) randomized controlled trials (RCTs); (2) the patients were over 18 years old; (3) the control group received the same amount of non-robotic therapy, such as usual care, conventional rehabilitation treatment, arm exercise, PT, OT, motor learning, self-guided therapy, task-oriented training, or home exercise program; the experimental group received RAT alone or RAT combined with additional treatments as a control group, for example in Hesse's study [31], patients in experimental group received RAT and individual arm therapy, and patients in control group only received individual arm therapy; (5) the results included at least one of the following measures: the Fugl-Meyer Upper Extremity (FM-UE), Barthel Index score (BI) or modified Barthel Index (mBI), Stroke Impact Scale (SIS), Frenchay Arm Test (FAT), ABILHAND Questionnaire and FIM for activity of daily living (ADL).

### Methodological quality and risk of bias assessment

One researcher evaluated the methodological quality and risk of bias of the included studies for random allocation, concealment of allocation, blinding of participants, personnel and assessors, incomplete outcome data, selective reporting and other bias with the Cochrane risk-of-bias tool [32]. If all of the above quality standards were of low risk, indicating the overall risk of bias was low and the methodological quality of study was high and considered as Grade A; if one or more of the standards were of high or unclear risk, the overall risk was moderate and the study was rated as Grade B; if none of the standards was of low risk, the overall risk was high and the study was rated as Grade C.

### Sensitivity analysis

We used the methodological features randomization produce, concealment of allocation, and blinding of assessors to test the robustness of the main results in a sensitivity analysis as described by Mehrholz [14] according to the instruction of the *Cochrane Handbook for Systematic Reviews of Interventions* [32]. We included trials with an adequate description of the randomization, a high quality of concealment of allocation and complete blinding of the assessors and analyzed the pooled effect of RAT on the outcomes of motor control and activity function.

### Data extraction

Two researchers extracted the following data from the included studies: the number of subjects; age, time after stroke; intervention protocols (frequency and duration, involved joint); comparison group; the primary outcome (FMA or FM-UE) measuring motor control; the secondary outcomes (FIM, SIS, BI, mBI, the ABILHAND Questionnaire and FAT) measuring the ADL according to a previous study [14]; and the mean differences and standard deviations (SDs) of the outcomes at the end of treatment and/or follow-up (≥ three months after treatment). When an included study compared RAT with two different non-robotic therapies (e.g., RAT versus usual care or versus enhanced upper limb therapy [13]) or discussed two different training methods of RAT (e.g., planar or planar with vertical training versus conventional rehabilitation [33]), we found that the results between the intervention groups and control groups differed significantly and therefore considered them to be two individual groups, according to previous studies [20,34]. If the study did not show detailed data of the primary outcome or secondary outcome, we would contact with the author for the raw data, if not available, the study was excluded.

### Data analysis

All data were recorded as the mean (SD). If the data were reported as 95% CI, the means and SDs were calculated using the appropriate statistical methods; if the data were reported as median/IQR, we conducted the author for data and calculated the mean, if the data were unavailable, the study was excluded. When the outcome was measured with the same scale, the mean difference was used; if not, the standard mean difference (SMD) was chosen to measure the effect [32]. Heterogeneity among studies was assessed using heterogeneity statistics (I^2^ statistic); P ≤ 0.1 and I^2^ ≥ 50% indicated significant heterogeneity[35]**.** The fixed-effects model was used when I^2^ < 50% or P > 0.1; if not (I^2^ ≥ 50% or P ≤ 0.1), the random-effects model was applied [36]. Four independent analyses were performed to evaluate the effect of RAT on upper-limb motor control and activity at the end of treatment and follow-up (≥ three months). Subgroup analyses were performed to investigate whether and how the poststroke phase and the training intensity (time per session × number of sessions, in hours) influenced the effect of RAT. There were no missing data in our study.

## Results

The search retrieved 502 articles. After removal of duplicate articles, 328 articles were screened, of which 260 articles were excluded. Sixty-two articles were assessed for eligibility, and forty-six studies were eligible for inclusion. The flow diagram of the study selection is shown in supplementary material (Additional file 1: Fig. S1).

### Characteristics of study

The study characteristics are described in Table 1. All included studies were RCTs published in 2010 to nowadays. The 46 included studies involved 2533 participants with a mean age ranging from 46.20 to 75.5 years old. Almost all (96.7%) patients had first-ever stroke, and 60% patients had ischemic stroke, 15.5% patients had hemorrhagic stroke, 40.7% patients had right hemiparesis, and 39.8% had left hemiparesis. The mean time poststroke ranged from 11 days to 8.5 years. The duration of RAT ranged from 10 days to 12 weeks, and the frequency ranged from two to ten sessions per week. The time spent engaged in RAT ranged from 30 to 180 min per session. The total number of RAT sessions ranged from 10 to 60. On average, patients received RAT four sessions per week for six weeks. The amount of treatment was presented using total time, and the cutoff time (15 h) was chosen according to a previous study in which the authors found that the difference in gains in FMA and FIM assessment between RAT and controls was not significant with a training time of 15 h and was significant with a training time of more than 15 h [10]. The control treatment group received the same amount of treatment as the intervention group. The arm robot used in the intervention group included the Mirror Image Movement Enabler (MIME), UL-EXO7, Amadeo Robotic System, InMotion ARM 2.0 Robot, Aremo Spring, Bi-Manu-Track, Myomo e100, Neuro-Rehabilitation Robot (NeReBot), electromyography (EMG)-driven robot, REJOYCE robot, Pneu-WREX, ReoGo system, and Gloreha robot, as described in Table 1. All included studies assessed motor control function with the FM-UE. Twenty-two studies assessed activity function using different measures, such as the FIM, SIS, BI and mBI.

### Methodological quality and risk of bias

We used the Cochrane risk-of-bias tool to assess the methodological quality of the involved studies. Additional file 2: Fig. S2 and Additional file 3: Fig. S3 presented the assessment of the risk of bias of all individual studies in detail. Forty studies (86.96%) described the randomization procedure, and six studies [37–42] did not show detailed information on random sequence generation. There were twenty-nine (63.04%) trials with adequate allocation concealment and thirty-eight (82.61%) trials with blinding of the assessors. However, only seven (15.22%) studies reported blinding of participations and personnel because the therapists who carried out the intervention can hardly be blinded to the group allocation. Table 2 showed the methodological quality of involved studies, only one included study [42] were rated as Grade C, and others were rated as Grade B.

### Meta-analysis

The outcomes of FM-UE (Additional file 4: Fig. S4) (SMD = 0.20, 95% CI 0.08 to 0.32, P = 0.001) and ADL (Additional file 6: Fig. S6) (SMD = 0.32, 95% CI 0.16 to 0.47, P < 0.0001) at the end-of-treatment were significantly higher in RAT group than controls, and the differences in outcomes of FM-UE (Additional file 5: Fig. S5) and ADL (Additional file 7: Fig. S7) between two groups were not found at the follow-up. Therefore, we pooled the outcomes of FM-UE and ADL at the end-of-treatment rather than at the follow-up in subgroup analyses. Additional file 8: Fig. S8 showed that there was no publication bias in those studies, sensitivity analysis (Additional file 9: Fig. S9, Additional file 10: Fig. S10, Additional file 11: Fig. S11, Additional file 12: Fig. S12) confirmed that the effect of RAT on the outcomes of the FM-UE and ADL at the end of treatment and follow-up was quite stable and not affected by the methodological quality.

### The amount of training

**Fig. 1:**
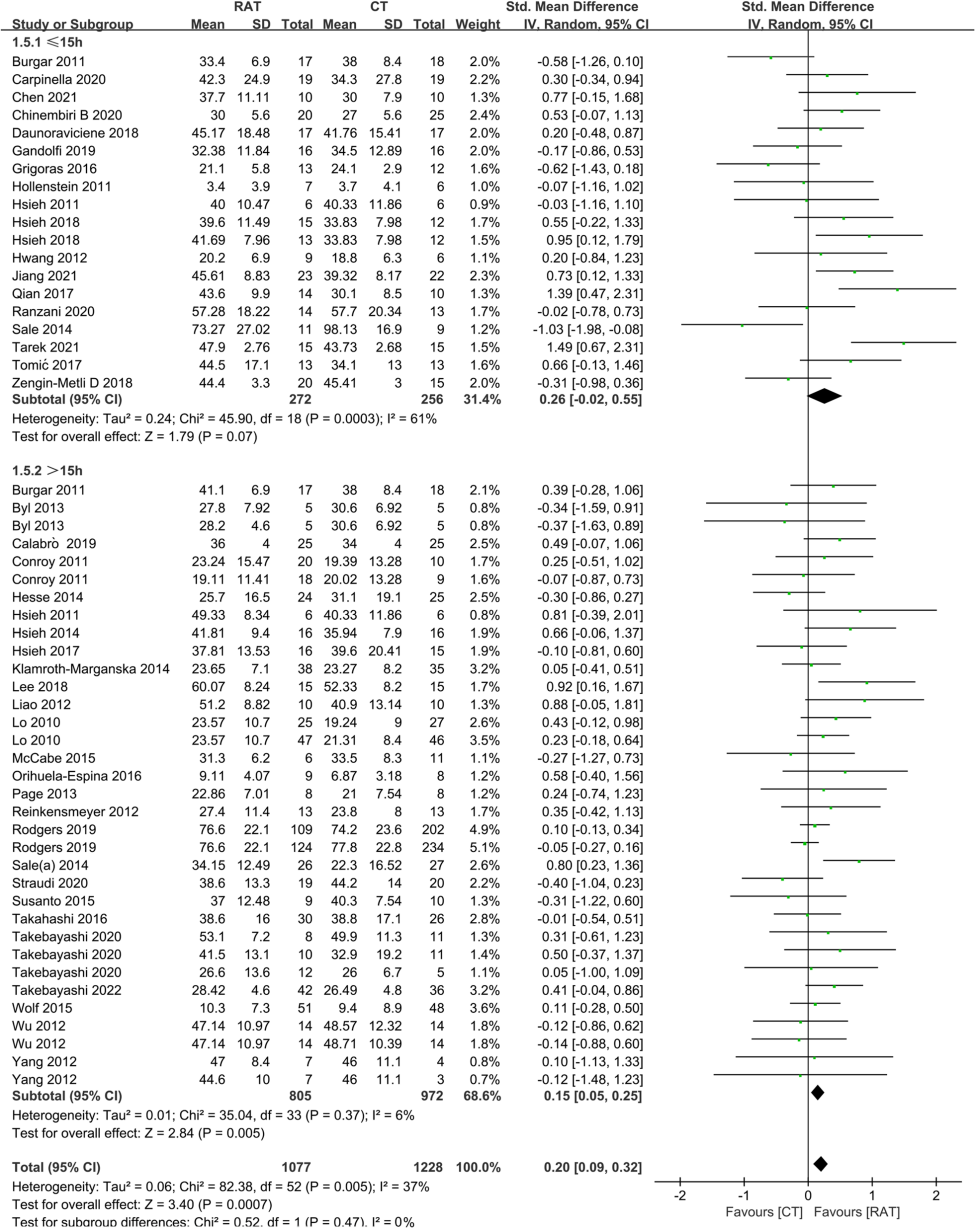
A subgroup analysis of the effect of RAT with different total training time versus non-robotic therapy on outcome of FM-UE at the end-of-treatment. The subgroup analysis showed that RAT better improved the outcomes of FM-UE at the end-of-treatment than controls when the total training time was more than 15 h (SMD = 0.15, 95% CI 0.05 to 0.25, P = 0.005), and had no significant clinical benefit with the total training time ≤ 15 h (SMD = 0.26, 95% CI − 0.02 to 0.55, P = 0.07)

**Fig. 2:**
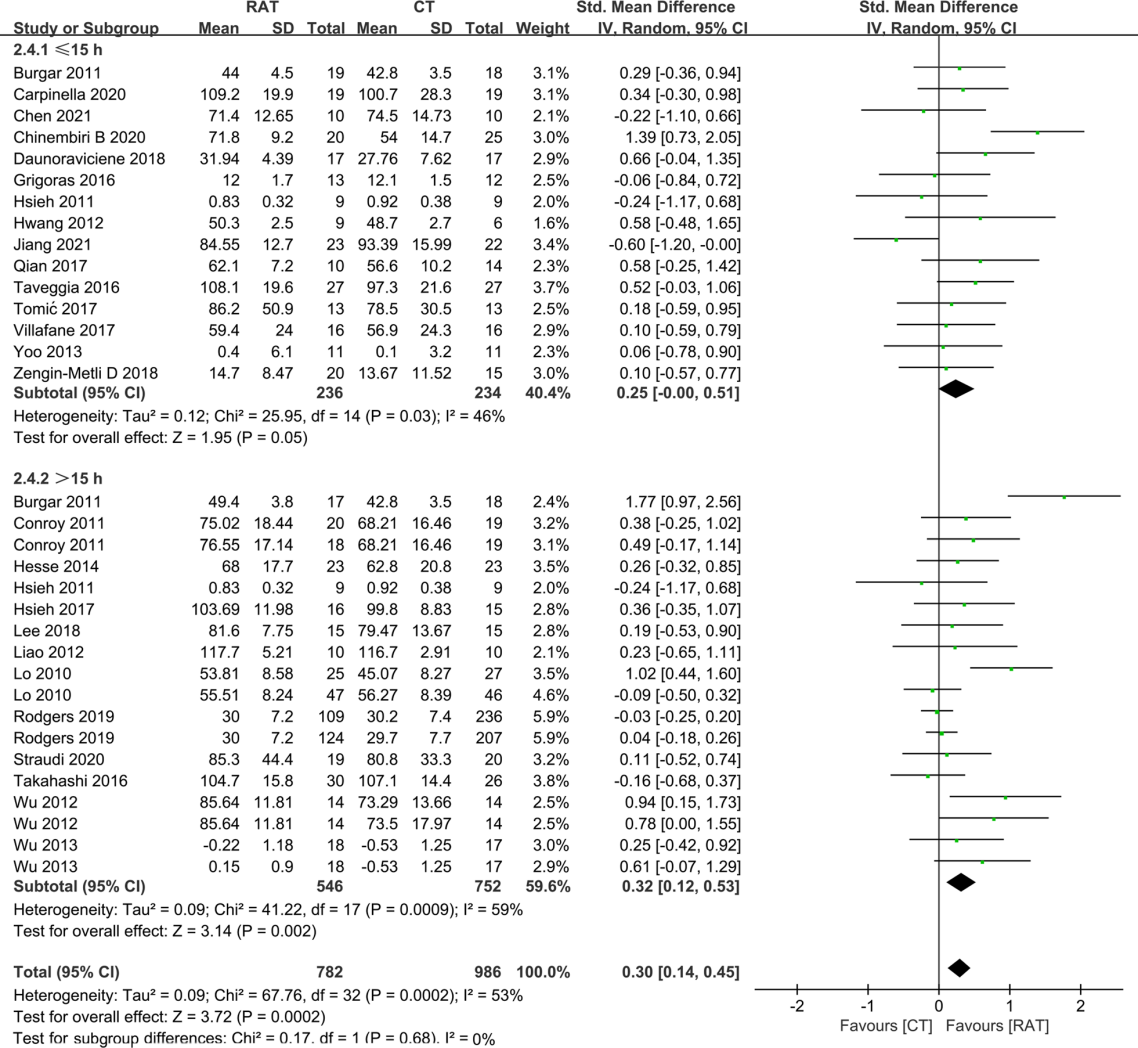
A subgroup analysis of the effect of RAT with different total training time versus non-robotic therapy on outcome of ADL at the end-of-treatment. The subgroup analysis indicated that RAT better improved the outcomes of ADL at the end-of-treatment than controls with the total training time more than 15 h (SMD = 0.32, 95% CI 0.12 to 0.53, P = 0.002), and had no additional benefit with the total training time ≤ 15 h (SMD = 0.25, 95% CI − 0.00 to 0.51, P = 0.05)

The amount of treatment was estimated by total time as described in a previous study [10,43]. We found that there was a statistically significant difference in the motor control results at the end of treatment between RAT and controls in the subset with a total time > 15 h (Fig. 1) (SMD = 0.15, 95% CI 0.05 to 0.25, P = 0.005), but no significant difference was found when the total time was ≤ 15 h (SMD = 0.26, 95% CI − 0.02 to 0.55, P = 0.07). A significant difference in outcome of activity function at the end of treatment between RAT and controls (Fig. 2) was also detected when the total time was more than 15 h (SMD = 0.32, 95% CI 0.12 to 0.53, P = 0.002), and no statistically significant difference was observed when the total time was ≤ 15 h. (SMD = 0.25, 95% CI − 0.00 to 0.51, P = 0.05).

### Level of impairment

**Fig. 3:**
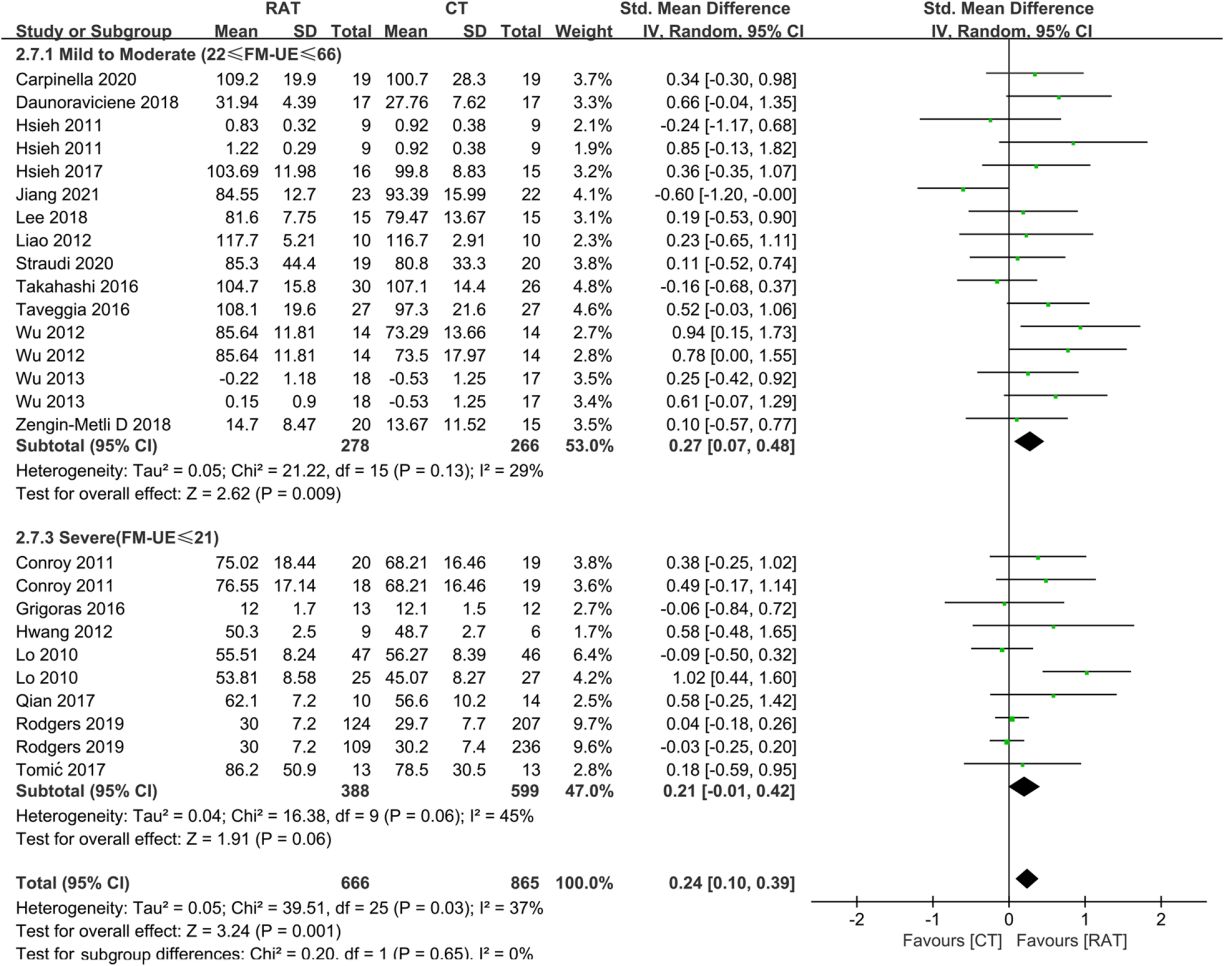
Comparison of the effect of RAT and non-robotic therapy on outcome of ADL at the end-of-treatment in patients with different level of impairment. The subgroup analysis showed that RAT significantly better improved the activity function in patients with mild to moderate paralysis (SMD = 0.27, 95% CI 0.07 to 0.48, P = 0.009), but had the same clinical effect as controls in patients with severe paralysis (SMD = 0.21, 95% CI − 0.01 to 0.42, P = 0.06)

The level of impairment was evaluated according to the baseline FM-UE scores, and the participants were classified into mild to moderate (22–66) and severe (≤ 21) groups as described in a previous study [29]. In the subgroup analysis, contrast with the study conducted by Wu [22], we found RAT significantly improved the FMA-UE scores at the end-of-treatment in the patients with mild-to-moderate paralysis, compared with controls (Additional file 13: Fig. S13) (SMD = 0.26, 95% CI 0.09 to 0.42, P = 0.002), and the difference between two groups at the end-of-treatment was not significant in patients with severe paralysis (SMD = 0.14, 95% CI − 0.01 to 0.30, P = 0.08). In line with the result of FM-UE, the between-group difference in outcome of ADL at the end-of-treatment was also observed in patients with mild to moderate paralysis (Fig. 3) (SMD = 0.27, 95% CI 0.07 to 0.48, P = 0.009) and was not found in patients with severe paralysis (SMD = 0.21, 95% CI − 0.01 to 0.42, P = 0.06).

### The training mode

**Fig. 4:**
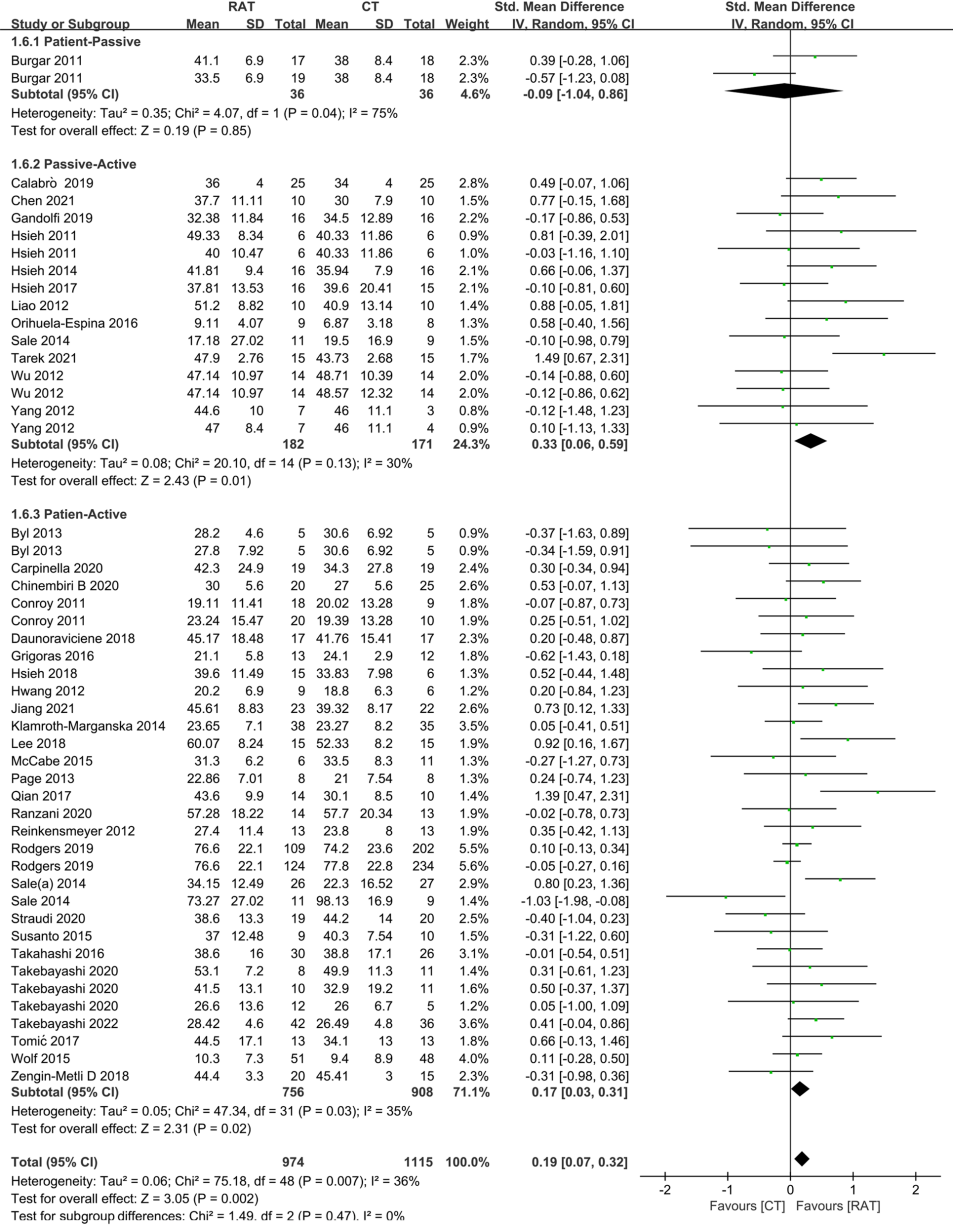
A subgroup analysis for the effect of RAT versus non-robotic therapy on outcome of FM-UE at the end-of-treatment in different training modes. The result indicated that RAT had better therapeutic effect on motor control function than controls when arm robots provide passive-active (SMD = 0.33, 95% CI 0.06 to 0.59, P = 0.01) and patient-active training (SMD = 0.17, 95% CI 0.03 to 0.31, P = 0.02)

**Fig. 5:**
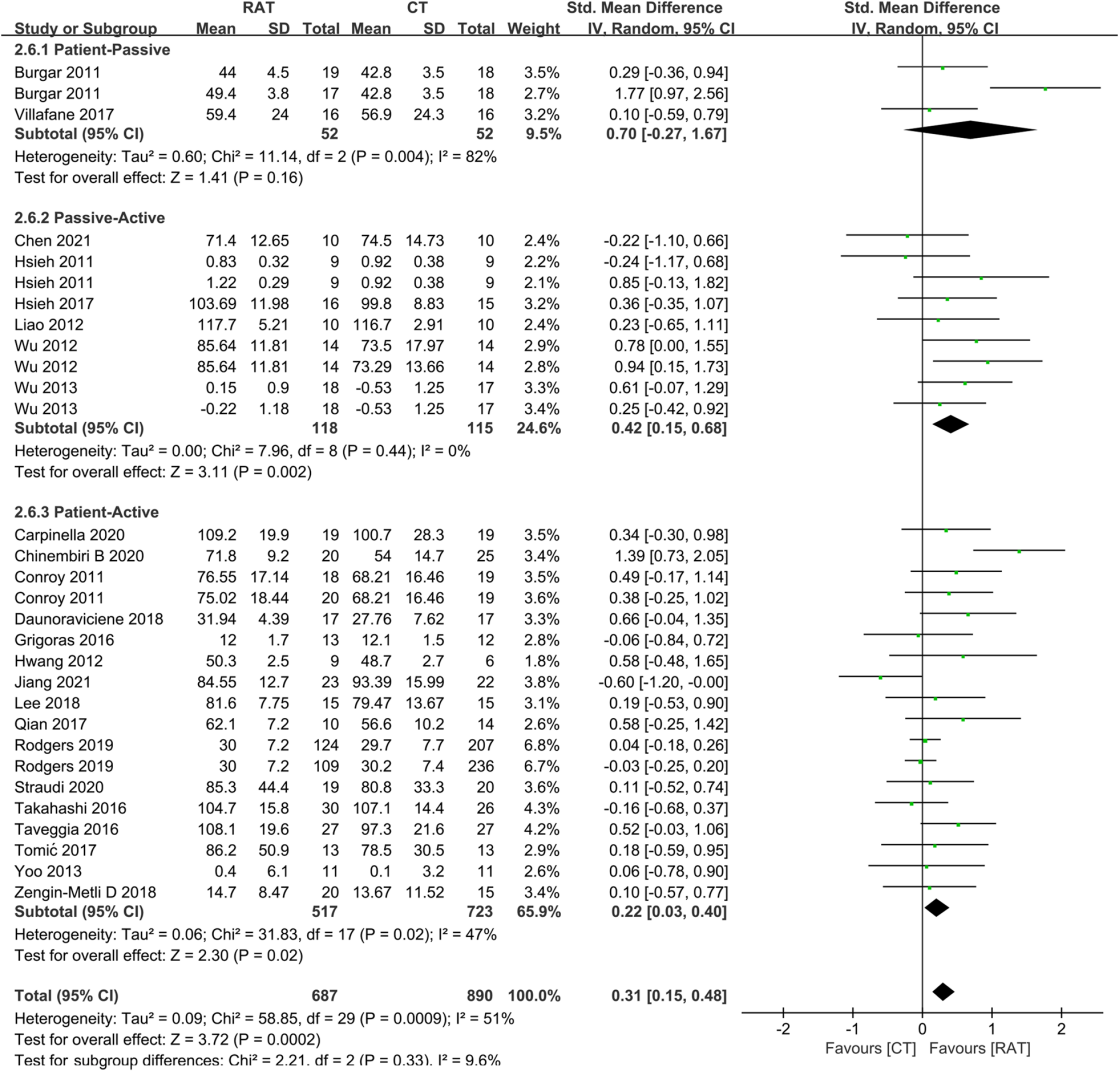
A subgroup analysis of the effect of RAT versus non-robotic therapy on outcome of ADL at the end-of-treatment in different training modes. The meta-analysis suggested that RAT could better improve the activity function than controls when arm robot provide passive-active (SMD = 0.42, 95% CI 0.15 to 0.68, P = 0.002) and patient-active training (SMD = 0.22, 95% CI 0.03 to 0.40, P = 0.02)

The training modes provided by the arm robots included patient-passive mode, patient-active mode and active resistance mode [44]. In the patient-active mode and active resistance mode, patients actively participate in the treatment, therefore, we considered them together as the patient-active group; while in several clinical trials, patients first received passive movement practice and then performed robot-assisted active tasks, thus, we considered them as the passive-active group. Figure 4 showed that the passive-active mode RAT group (SMD = 0.33, 95% CI 0.06 to 0.59, P = 0.01) and the patient-active mode RAT group (SMD = 0.17, 95% CI 0.03 to 0.31, P = 0.02) had the higher outcome of the FM-UE at the end of treatment, compared with control group; while the patient-passive mode RAT group (SMD = -0.09, 95% CI -1.04 to 0.86, P = 0.85) had the same outcome of the FM-UE as control group. The outcome of the ADL at the end-of-treatment was also significantly higher in the passive-active mode RAT group (Fig. 5) (SMD = 0.42, 95% CI 0.15 to 0.68, P = 0.002) and patient-active mode RAT group (Fig. 5) (SMD = 0.22, 95% CI 0.03 to 0.40, P = 0.02) compared to controls, and the difference in outcome of the ADL between RAT and control groups was not significant when RAT was applied in the patient-passive mode (SMD = 0.70, 95% CI -0.27 to 1.67, P = 0.16).

## Discussion

This systematic review demonstrated that RAT has the immediate benefits on motor control and activity function compared with non-robotic therapy. Moreover, we found the superiority of RAT in improving motor control and activity function was observed when it was supplied in passive-active mode or patient-active mode, with the amount of training more than 15 h and to patients with mild to moderate impairment.

In our study, we found that RAT could better improve the outcomes of the FM-UE and the activity function at the end-of-treatment compared with controls. Several reasons might account for this result. First, arm robots can simultaneously provide highly repetitive, interactive forms of training and multisensory stimulation for the paretic limb [45], and several robots can provide gravity support for the upper limb, allow patients to perform a complete functional movement with their own effort. Additionally, some arm robots can precisely assess the limb function such as interaction forces, range of motion and limb movement reports, and then provide biofeedback, thus increasing the objective of training and promoting recovery of motor control of the upper limb after stroke [46].

The differences in outcomes of the FM-UE and ADL between RAT and controls were significant at the end-of-treatment, but were not in the follow-up period, indicating the long-term effect of RAT was not better than controls. Consistent with our study, Masiero [47] and Susanto [48] conducted follow-up studies and found that although RAT could improve the FM-UE, the differences between RAT and control groups were nonsignificant. However, the small sample size (n = 11/n = 7) in our study might cause our result underpowered, the future research involving a larger sample is needed to investigate the long-term effect of RAT.

Considering the optimal total training time of RAT, this meta-analysis suggested that a larger amount (> 15 h) of RAT could better improve the motor control and activity compared with controls. In our study, we found that the differences in outcomes of the FM-UE and ADL between RAT and controls were significant when the total training time was more than 15 h and not significant when training time was less than 15 h, in consistent with a previous study [30] in which the authors found that the gains in the FMA and FIM were not different between the RAT and control group when the total training time was 15 h. Sehle’s [49] study found that RAT led to the higher motor excitability compared with control treatment, and the motor excitability was positively correlated with the amount of robot-assisted training. We speculate that when total training time is less than 15 h, the motor excitability induced by RAT is weak and couldn't successfully translate to clinical improvements, and the motor excitability becomes stronger enough to translate into clinical improvement with the total training time increasing.

The movement practice and application of robotic force are two interacting processes of RAT, and which process is more beneficial is controversial. A previous study [26] found that robotically finishing a movement for a patient with stroke did not show better improvement of function than usual movement practice, and using robotic forces to assist patients to complete correct movements could focus and intensify patients' effort and attention to the treatment, achieving better outcomes [50]. Active participation of the patients is critical for neuroplasticity, motor learning and rehabilitation [50,51], and studies have found that rehabilitation treatment integrated with patients' voluntary movement could facilitate the recovery of lost motor ability [16,52]. The level of patients' active participation is partially influenced by the control systems of robots and the paralysis level of patients. The control systems of robots can be roughly divided into patient-passive control and patient-active control [16]. Arm robots implementing patient-passive control are suitable for patients with severe paralysis, and provide passive mode training for them to passively execute repetitive movement along predefined trajectories, and the active participation of patients is often neglected during such patient-passive training mode [53]. Robots equipped with patient-active control, such as patient-cooperative control, assist-as-needed control, impedance-based control and EMG-signal-based control, can regulate the human–robot interaction based on the motion intention and disability level of patients [54], and the training modes provided by those patient-active controls include passive mode for patients with severe disability, active mode and active-resistance mode. In our study, we found that RAT could better improve motor and activity function in patients with mild to moderate impairment than controls, and RAT had the same effect as controls in patients with severe impairment. RAT showed significant benefits for motor control and activity compared with controls when it provided patient-active and passive-active training, whereas RAT had the similar effects with controls when it provided patient-passive training. As we known, patients with severe paralysis perform few voluntary movements in the treatment, indicating decreased active participation, and patients might pay more attention and effort in the patient-active and passive-active training than passive training, therefore, the above findings in our study demonstrated that the better therapeutic beneficial effect of arm robots might not result from providing passive automatic movement but mainly from assisting patients to complete voluntary movements, and the higher degree of patients' active participation cause better improvement in motor and activity function.

Even though there were significant differences in the outcomes of the FM-UE and ADL at the end of the intervention between RAT and controls, the overall effect size was small or medium in some subgroups, indicating that the beneficial therapeutic effect of arm robots was limited, which suggested that the clinical application must be used with caution regarding the amount of treatment, the impairment level of patients, and the training mode. In addition, almost all (96.7%) patients in our study had first-ever stroke, and the majority (60%) of them suffered from ischemic stroke; hence, the results might not be applicable for patients with recurrent stroke or hemorrhagic stroke.

There were several limitations in this meta-analysis and review as following: (1) As we known, the application of arm robot such as arm robot alone or RAT combined with controls may affect the differences in outcomes of motor control and activity between intervention and control group, but we have not further discussed this factor; (2) We only investigated the effect of total training time on effectiveness of RAT, however other parameters such as the number of repetitions, frequency and duration of RAT also influence its effect; (3) The small sample size in follow-up group may cause our results underpowered.

## Conclusion

Our study suggest that RAT has the significant immediate beneficial effects on motor control and activity function of hemiparetic upper limb in patients after stroke, but there is no evidence to support its long-term effect. The superiority of RAT is influenced by the amount of training time, the training mode and the impairment level of patients. To achieve the best therapeutic effect, arm robots should be applied with training time more than 15 h, in patient-active mode or passive-active mode for patients with mild to moderate impairment.

Considering the application of arm robot, the number of repetitions, the frequency and the duration of robot-assisted training may also influence the effectiveness of RAT, future study should stratify the patients according to the those factors to further determine the optimal application and parameters of RAT.

**Table 1:** The characteristic of included studies

Study	No. of participants (Exp/Ctr)	Mean age (years) (Exp/Ctr) (mean/SD)	Mean time Post-stroke (Exp/Ctr)	Intervention (Robot)	Duration and frequency	Joint involved	Control group	Outcome measures
Burgar (2011)	25(17/18)	60(2)/68(3)	17(3)/ 11(1)(d)	RAT (MIME)	1 h/session, 5 sessions/week for 3w	Whole arm	CT	Function: FM-UE/WMFT Activity: FIM Assessment: after-treatment
Burgar (2011)	25(17/18)	60(2)/68(3)	17(3)/ 11(1)(d)	RAT (MIME)	1 h/session, 10 sessions/week for 3w	Whole arm	CT	Function: FM-UE/WMFT Activity: FIM Assessment: after-treatment
Byl (2013)	18(5/5/)	59.6(14.6)	8.5(4.5)(y)	Unilateral RAT (UL-EXO7)	90 min/session, 2 sessions/week for 6w	Whole arm	PT	Function: FM-UE Assessment: after-treatment
Byl (2013)	18(5/5)	59.6(14.6)	8.5(4.5)(y)	Bilateral RAT (UL-EXO7)	90 min/session, 2 sessions/week for 6w	Whole arm	PT	Function: FM-UE Assessment: after-treatment
Calabrò (2019)	50(25/25)	64(3)	10(2)(m	RAT (Amadeo)	45 min/session, 5 sessions/week for 8w	Hand	PT	Function: FM-UE/NHPT Assessment: after-treatment
Carpinella (2020)	40(19/19)	65(9.61)/58(18.42)	≥ 6 months	RAT (Braccio di Ferro)	45 min/session, 5 sessions/week for 4w	Shoulder and elbow	PT	Function: FM-UE/ FM-UE(proximal)/ FM-UE(distal) Activity: FIM Assessment: after-treatment
Chen (2021)	20(10/10)	46.20(7.02)/48.60(9.95)	97.10(84.37)/86.40(61.92) (d)	RAT(Armule)	45 min/day, 5d/w for 4w	Whole arm	Cognitive and Occupational rehabilitation	Function: FM-UE/ Activity: mBI Assessment: after-treatment
Conroy(2011)	62(20/21)	57.8(10.7)	4.2(5.48)(y)	Robot-assisted planar treatment (InMotion ARM2.0)	60 min/session, 3 sessions/week for 6w	Whole arm	Conventional arm exercise	Function: FM-UE/WMFT Activity: FIM Assessment: after-treatment and 3-month follow-up
Conroy (2011)	62(21/21)	57.8(10.7)	4.2(5.48)(y)	Robot-assisted planar and vertical treatment (InMotion ARM2.0)	60 min/session, 3 sessions/week for 6w	Whole arm	Conventional arm exercise	Function: FM-UE/WMFT Activity: FIM Assessment: after-treatment and 3-month follow-up
Chinembiri.B (2020)	60(30/30)	57.72(7.37)/57.25(9.23)	Acute	RAT + OT (Fourier M2)	70 min (20minRAT + 50minOT)/day, 5 days/week for 6w	Whole arm	OT	Function: FM-UE Activity: BI Assessment: after-treatment
Daunoravicien (2018)	34(17/17)	65.88(4.87)/65.47(4.05)	8.6(3.53)/9.65(6.18) (w)	RAT (Armeo Spring)	30 min/day, 5 days/week for 2w	Whole arm	OT	Function: FM-UE/MAS/ ROM Activity: FIM Assessment: after-treatment
Gandolfi (2019)	32(16/16)	59.31(14.40)/59.13(14.97)	6.0(3.1)/5.1(2.2)(y)	RAT (Armotion)	45 min/session, 2 sessions/week for 5w	Shoulder and elbow	Conventional treatment	Function: FMA/MRC/MAS Assessment: after-treatment
Grigoras(2016)	25(13/12)	63(9)/65(11)	4(1) months	RAT (NMES-robot)	30 min/session, 10–12 session	Whole arm	Standard arm therapy	Function: FM-UE/BBT Activity: SIS Assessment: after-treatment
Hesse (2014)	50(25/25)	71.4(15.5)/ 69.7(16.6)	4.5(1.7)/4.5(1.4)(w)	RAT + individual arm therapy (Bi-ManuTrack)	30 min (RAT) + 30 min (individual arm therapy)/day, 5 days/week for 4w	Whole arm	Double sessions of individual arm therapy	Function: FM-UE/MRC/ BBT Activity: FIM Assessment: after-treatment and 3-month follow-up
Hollenstein (2011)	13(7/6)	54(12)/56(11)	> 12 months	RAT (NA)	30 min/session, 5 session/week for 2w	Whole arm	CT	Function: FM-UE Assessment: after-treatment
Hsieh (2011)	18(6/6/6)	54(8)	17(7)/28(20) (m)	Higher-intensity RT (Bi-Manu-Track)	90–105 min/session, 5 session/week for 4w	Whole arm	Occupational therapy	Function: FM-UE/MRC/ MAL Assessment: after-treatment
Hsieh (2011)	18(6/6/6)	54(8)	17(7)/28(20) (m)	Lower-intensity RT (Bi-Manu-Track)	90–105 min/session, 5 session/week for 4w	Whole arm	Occupational therapy	Function: FM-UE/MRC/MAL Assessment: after-treatment
Hsieh (2014)	48(32/16)	53(10)/54(10)	22(14)/28(19) (m)	RT (Bi-Manu-Track)	90–105 min/session, 5 session/week for 4w	Whole arm	Occupational therapy	Function: FM-UE/MRC/MAL Assessment: after-treatment
Hwang (2012)	17(9/6)	50.6(10.0)	6.5(5.3)(m)	2 weeks RAT + 2 weeks passive therapy (Amadeo)	45 min/session, 5 sessions/week, 4w	Hand and finger	Passive therapy	Function: NHPT/FM-WH/ FM-proximal arm Assessment: after-treatment
Hsieh (2017)	31(16/15)	49.28(10.90)/52.87(10.40)	2.56(1.69)/ 2.21(1.11)(m)	RAT + task-oriented approach (Bi-Manu-Track)	90 min/session, 5 session/week for 4w	Whole arm	Task-oriented approach	Function: FM-UE/BBT/Grip Activity: FIM/SIS Assessment: after-treatment
Hsieh (2018)	44(15/18)	54.42	20.58(m)	Proximal-emphasized robotic rehabilitation (InMotion 2.0)	90–100 min/day, 5 days/week, 4w	Shoulder and elbow	Conventional rehabilitation	Function: FM-UE/ FM-UE(pro)/FM-UE(dis) /MRC Assessment: after-treatment
Hsieh (2018)	44(13/18)	54.42	20.58(m)	Distal-emphasized robotic rehabilitation (InMotion 2.0)	90–100 min/day, 5 days/week, 4w	Shoulder and elbow	Conventional rehabilitation	Function: FM-UE/ FM-UE(pro)/FM-UE(distal)/ MRC Assessment: after-treatment
Jiang (2021)	45 (23/22)	62.43(11.29)/66(11.51)	20.09(5.53)/19.41(7.04)(d)	RAT (ArmeoR Spring arm robot)	30 min/session, 2 session/d for 10d	Whole arm	Conventional rehabilitation	Function: FM-UE/ Activity: FIM/BI Assessment: after-treatment and follow-up (1 month)
Klamroth-Marganska (2014)	77(39/38)	55(13)/58(14)	52(44)/ 40(45)(m)	RAT (ARMin)	45 min/session, 3 session/week, 8w	Whole arm	Conventional therapy	Function: FM-UE/MAS/ WMFT Activity: SIS Assessment: after-treatment
Lee (2018)	30(15/15)	52.07(14.07)/50.27(11.17)	≥ 7 months	RAT + OT (REJOYCE robot)	30 min RAT + 30 min OT/session 5 sessions/week,8w	Whole arm	60 min OT	Function: FM-UE Activity: BI Assessment: after-treatment
Liao (2012)	20(10/10)	55.51(11.17)/54.56(8.20)	23.90(13.39)/ 22.20(17.47)(m)	RAT (Bi-Manu-Track)	90–105 min/session, 5 session/week,4w	Whole arm	Dose-matched active control therapy	Function: FM-UE Activity: FIM Assessment: after-treatment
Lo (2010)	127(49/28)	64.6(11.3)	4.7(4.3)(y)	RAT (NA)	1 h/session, 3 session/week,12w	Whole arm	Usual care	Function: FM-UE/MAS/ WMFT Activity: SIS Assessment: after-treatment
Lo (2010)	127(49/50)	64.6(11.3)	4.7(4.3)(y)	RAT (NA)	1 h/session, 3 session/week,12w	Whole arm	Comparison rehabilitation treatment	Function: FM-UE/MAS/ WMFT Activity: SIS Assessment: after-treatment
McCabe (2015)	25(12/13)	N/A	> 1 year	1.5 h RAT + 3.5 h motor learning (InMotion2)	5 h/day, 5 days/week, 12w (60session)	Shoulder and elbow	5 h motor learning	Function: FM-UE/AMAT Assessment: after-treatment
Orihuela-Espina (2016)	17(9/8)	56.22(13.72)/55.00(25.78)	2.18(1.25)/ 2.44(0.88)(m)	RAT (Amadeo)	40 min/session, 5 sessions/week,8w	Hand	OT	Function: FM-hand/MI Assessment: after-treatment
Page (2013)	16(8/8)	57.0(11.02)	75.0(87.63) (m)	Robot-assisted task-specific practice (Myomo e100)	1 h/day, 3 days/week, 8w	Elbow	Task-specific practice	Function: FM-UE Activity: SIS Assessment: after-treatment
Qian. (2017)	24(14/10)	54.6(11.3)/ 64.6(3.43)	Subacute	NMES-robotic arm	40 min/session, 5 session/week, 4w	Whole arm	Time-matched traditional therapy	Function: FMA/MAS/ ARAT Activity: FIM Assessment: after treatment and 3-month follow-up
Ranzani. (2020)	27(14/13)	70.00(12.79)/67.46(11.39)	3.14(1.51)/ 3.08(1.32)(w)	RAT (ReHapticKnob)	45 min/day for 15 days	Hand	Conventional neurocognitive therapy	Function: FMA Assessment: after treatment and 8-months follow-up
Reinkensmeyer (2012)	27(13/14)	60(10)/61(13)	65(47)/67(56) (m)	RAT (Pneu-WREX)	1 h/session, 3sessions/week, 8w (24sessions)	Whole arm	Conventional therapy	Function: FM-UE/ Grip strength/BBT Assessment: after-treatment
Rodgers (2019)	770(257/254)	61(14)	42.8(46.6)(w)	RAT (MIT-Manus)	45 min/session, 3 sessions/week,12w	Whole arm	Usual care	Function: FM-UE/ARAT/ Activity: SIS/BI Assessment: after-treatment and 3-month follow-up
Rodgers (2019)	770(257/259)	61(14)	42.8(46.6)(w)	RAT (MIT-Manus)	45 min/session, 3 sessions/week,12w	Whole arm	Enhanced upper limb therapy	Function: FM-UE/ARAT Activity: BI/SIS Assessment: after-treatment and 3-month follow-up
Sale (a) (2014)	53(26/27)	67.7(14.2)	30(7)(d)	RAT (MIT-Manus)	5 session/week, 6w	Shoulder and elbow	Conventional therapy(pt)	Function: FM-UE/ MAS-S/MAS-E/MI/pROM Assessment: after-treatment
Sale (2014)	20(11/9)	72.56(8.98) 67.0(12.4)	30(7)(d)	RAT (Amadeo Robotic System)	40 min/session, 5 session/week,4w	Hand	OT	Function: FM-UE/MI/MRC/BBT Assessment: after-treatment and 3-month follow-up
Straudi (2020)	39(19/20)	66.2(11.5)	39.5(30)(d)	RAT + FES (ReoGo therapy systerm)	100 min/session, 5 session/week, 6w	Whole arm	Intensive conventional Therapy	Function: FM-UE/MAS/BBT/ WMFT Activity: BI Assessment: after-treatment and 6-month Assessment
Susanto (2015)	19(9/10)	53.2(9.9)	16.4(5.8)/ 16.1(5.1)(m)	RAT (EMG-driven hand robot)	1 h/session, 3–5 sessions/week, 20 sessions in 6w	Hand	Non-robotic therapy	Function: FM-SE/ FM-WH/FM-total /WMFT-FT/ARAT Assessment: after-treatment and 6-month follow-up
Takahashi (2016)	60(30/30)	65.2(10.9)/ 64.6(11.5)	47.8(7.0)/46.9(8.1)(d)	RAT (ReoGo system)	40 min/session,7 sessions/week, 6w	Whole arm	Self-guided therapy	Function: FMA/MI/WMFT Assessment: after-treatment
Takabayashi (2020)	60(30/30)	63(10.8)	47.7(5.6)(d)	RAT (ReoGo system)	40 min/session, 5 session/ week for 6w	Whole arm	Self-guided therapy	Function: FM-UE/ARAT Activity: SIS Assessment: after-treatment
Takabayashi (2022)	78(42/36)	59.0(12)/58.0(10)	≥ 6 months	RAT (ReoGo-J)	1 h/session, 3 session/week, for 10w	Whole arm	Self-training + usual care	Function: FM-UE Assessment: after-treatment
Tarek (2021)	45(30/15)	57.26(4.66)/58.66(4.65)	≥ 6 months	RAT (Amadeo robotic system)	1 h/session, 3 session/week for 4 w	Whole arm	PT	Function: FM-UE Assessment: after-treatment
Taveggia (2016)	54(27/27)	73(10)/68(13)	47.8(7.0)/ 46.9(8.1)(d)	RAT (Armeo)	30 min/session, 5 session/ week, 6w	Whole arm	Conventional treatment	Function: MI Activity: FIM(ACTIVITY) Assessment: after-treatment
Tomić (2017)	26(13/13)	56.5(7.4)/ 58.5(5.2)	35.5(9.7)/37.3(7.7)(d)	RAT (ArmAssist)	30 min/session, 5 session/week for 3w	Arm support	PT and OT	Function: FM-UE/WMFT Activity: BI Assessment: after-treatment
Villafañe (2017)	32(16/16)	67(11)/70 (12)	Early subacute phase	RAT + OT/PT (Gloreha)	30 min RAT + 30 min PT and OT, 3d/week for 3w	Hand	PT and OT	Function: NIHSS/ MAS/ MI/ Activity: BI Assessment: after-treatment
Wolf (2015)	96(48/48)	59(14)/55(12)	116(53)/127(46)(d)	Home-based robotic-assisted device(HEP) (Hand Mentor Pro)	3 h/day, 5 d/week for 8w	Whole arm	Home exercise program	Function: FM-UE/WMFT Activity: SIS Assessment: after-treatment
Wu (2012)	28(14/14)	54.49(9.69)	17.62(10.50) (m)	RAT (Bi-Manu-Track)	90–105 min/session, 5 sessions/week,4w	Hand	PT	Function: FM-UE Activity: SIS Assessment: after-treatment
Wu (2013)	53(18/18/17)	54.95(9.90)/54.22(9.78)	19.00(15.51)/ 23.41(15.24) (m)	Unilateral RAT (Bi-Manu-Track)	90–105 min/day, 5 days/week, 4w	Forearm	Conventional therapy	Function: WMFT Activity: the ABILHAND Questionnaire Assessment: after treatment
Wu (2013)	53(18/18/17)	52.21(12.20)/54.22 (9.78)	23.28(15.37)/ 23.41(15.24) (m)	Bilateral RAT (Bi-Manu-Track)	90–105 min/day, 5 days/week, 4w	Forearm	Conventional therapy	Function: WMFT Activity: the ABILHAND Questionnaire Assessment: after treatment
Yang (2012)	21(7/7)	51.3(8.24)	13.8(5.7)(m)	Unilateral RAT (Bi-Manu-Track)	90–105 min/session, 5 sessions/week, 4w	Forearm and wrist	Standard rehabilitation	Function: FMA-UE/MRC/Grip strength Assessment: after-treatment
Yang (2012)	21(7/7)	51.3(8.24)	13.8(5.7)(m)	Bilateral RAT (Bi-Manu-Track)	90–105 min/session, 5 sessions/week, 4w	Forearm and wrist	Standard rehabilitation	Function: FMA-UE/MRC/Grip strength Assessment: after-treatment
Yoo (2013)	22(11/11)	51(11)/50(9)	46(42)/42(33)(m)	3-dimensional RAT + CT (Reogo system)	30 min RAT + 60 min CT, 3d/week for 6w	Whole arm	CT	Function: WMFT/BBT Activity: mBI Assessment: after-treatment
Zengin-Metli D (2018)	35(20/15)	63.27(3.88)/59.25(8.10)	11.33(5.26)/ 10.7(4.9)(w)	RAT (Armeo)	30 min/session, 5 session/week for 3w	Whole arm	Rehabilitation program	Function: FM-UE Activity: FIM Assessment: after-treatment

*FM-UE* Fugl-Meyer Upper Extremity, *WMFT* Wolf Motor Function Test, *FIM* Functional Independence Measure, *NHPT* Nine Hole Peg Test, *mBI* modified Barthel Index, *BI* Barthel Index, *MAS* Modified Ashworth Scale, *ROM* Range of movement, *MRC* Medical Research Council Scale, *SIS* Stroke Impact Scale, *BBT* Box & Block Test, *MAL* Motor Activity Log, *FM-WH* FM-wrist and hand, *AMAT* Arm Motor Ability Test, *MI* Motricity Index, *ARAT* Arm Motor Ability Test

**Table 2:** The methodological quality assessment of included studies

Study	Random sequence generation		Allocation concealment	Blinding of participants and personnel	Blinding of outcome assessment	Incomplete outcome data	Selective reporting	Other bias	Grade
Burgar (2011)	Low risk	Unclear		High risk	Unclear	Unclear	Unclear	Unclear	B
Byl (2013)	Low risk	Low risk		High risk	Low risk	Low risk	Low risk	Unclear	B
Calabrò (2019)	Low risk	Low risk		Unclear	Low risk	Low risk	Low risk	Unclear	B
Carpinella (2020)	Low risk	Low risk		Low risk	Unclear	Low risk	Low risk	Unclear	B
Chen. (2021)	Low risk	Low risk		High risk	Low risk	Low risk	Unclear	Unclear	B
Chinembiri.B (2020)	Low risk	Low risk		High risk	Low risk	Low risk	Low risk	Unclear	B
Conroy (2011)	Low risk	Unclear		High risk	Low risk	Low risk	Low risk	Unclear	B
Daunoravicien (2018)	Low risk	Low risk		Low risk	Low risk	Low risk	Low risk	Unclear	B
Gandolfi (2019)	Low risk	Low risk		High risk	Low risk	Unclear	Low risk	Unclear	B
Grigoras (2016)	Unclear	Unclear		High risk	High risk	Low risk	Low risk	Unclear	B
Hesse (2014)	Low risk	Unclear		Unclear	Low risk	Low risk	Low risk	Unclear	B
Hollenstein (2011)	Low risk	Unclear		Unclear	Unclear	Low risk	Unclear	Unclear	B
Hsieh (2011)	Low risk	Low risk		Low risk	Low risk	Low risk	Low risk	Unclear	B
Hsieh (2014)	Low risk	Unclear		High risk	Low risk	Low risk	Unclear	Unclear	B
Hsieh (2017)	Low risk	Low risk		Unclear	Low risk	Low risk	Low risk	Unclear	B
Hsieh (2018)	Low risk	Low risk		Unclear	Low risk	Low risk	Low risk	Unclear	B
Hwang (2012)	Low risk	Low risk		High risk	Low risk	Low risk	Low risk	Unclear	B
Jiang. (2021)	Low risk	Unclear		Unclear	Unclear	Unclear	Unclear	Unclear	C
Klamroth-Marganska (2014)	Low risk	Unclear		Unclear	Low risk	Low risk	Low risk	Unclear	B
Lee (2018)	Low risk	Low risk		Unclear	Unclear	Low risk	Low risk	Unclear	B
Liao (2012)	Low risk	Low risk		Unclear	Low risk	Low risk	Low risk	Unclear	B
Lo (2010)	Low risk	Unclear		High risk	Low risk	Unclear	Low risk	Unclear	B
McCabe (2015)	Low risk	Low risk		Unclear	Low risk	Low risk	Low risk	Unclear	B
Orihuela-Espina (2016)	Low risk	Low risk		High risk	High risk	Unclear	Low risk	Unclear	B
Page (2013)	Low risk	Low risk		High risk	Low risk	Low risk	Low risk	Unclear	B
Qian (2017)	Low risk	Unclear		Low risk	Low risk	Low risk	Unclear	Unclear	B
Ranzani. (2020)	Low risk	Unclear		High risk	Low risk	Low risk	Unclear	Unclear	B
Reinkensmeyer (2012)	Low risk	Low risk		Unclear	Low risk	Low risk	Low risk	Unclear	B
Rodgers (2019)	Low risk	Low risk		High risk	Low risk	Low risk	Low risk	Unclear	B
Sale (a) (2014)	Low risk	Low risk		Low risk	Low risk	Unclear	Low risk	Unclear	B
Sale (2014)	Low risk	Low risk		Low risk	Low risk	Low risk	Low risk	Unclear	B
Straudi (2020)	Low risk	Low risk		Unclear	Low risk	Low risk	Low risk	Unclear	B
Susanto (2015)	Low risk	Low risk		Unclear	Low risk	Unclear	Low risk	Unclear	B
Takahashi (2016)	Low risk	Low risk		Unclear	Low risk	Low risk	Low risk	Unclear	B
Takabayashi (2020)	Low risk	Unclear		Unclear	Unclear	Low risk	Low risk	Unclear	B
Takabayashi (2022)	Low risk	Low risk		High risk	Low risk	Unclear	Unclear	Unclear	B
Tarek (2021)	Unclear	Unclear		Unclear	Unclear	Unclear	Unclear	Unclear	C
Taveggia (2016)	Low risk	Low risk		Unclear	Low risk	Unclear	Low risk	Unclear	B
Tomić (2017)	Low risk	Low risk		Unclear	Low risk	Low risk	Low risk	Unclear	B
Villafañe (2017)	Unclear	Unclear		High risk	Low risk	Low risk	Unclear	Unclear	B
Wolf (2015)	Low risk	Unclear		Unclear	Low risk	Low risk	Unclear	Unclear	B
Wu (2012)	Low risk	Low risk		High risk	Low risk	Low risk	Low risk	Unclear	B
Wu (2013)	Unclear	Low risk		Low risk	Low risk	Low risk	Low risk	Unclear	B
Yang (2012)	Low risk	Low risk		Unclear	Low risk	Unclear	Low risk	Unclear	B
Yoo (2013)	Unclear	Unclear		High risk	Low risk	Low risk	Unclear	Unclear	B
Zengin-Metli. (2018)	Unclear	Unclear		Unclear	Low risk	Low risk	Unclear	Unclear	B

## Supplementary Information

**Additional file 1: Fig S1.** Flow diagram of study selection.

**Additional file 2: Fig S2.** Risk of bias summary for all included studies.

**Additional file 3: Fig S3.** Risk of bias graph for all included studies.

**Additional file 4: Fig S4.** Comparison of the effect of RAT and non-robotic therapy on outcome of FM-UE scale at the end-of-treatment. The result showed that RAT had the additional immediated benfits on motor control compared with controls (SMD = 0.20, 95% CI 0.08 to 0.32, P = 0.001).

**Additional file 5: Fig 5.** Comparison of the effect of RAT and non-robotic therapy on outcome of FM-UE at the follow-up (≥ 3 months). The result showed that the long-term effect of RAT on motor control was same as controls (SMD = -0.07, 95% CI -0.21 to 0.07, P = 0.31).

**Additional file 6: Fig S6.** Comparison of the effect of RAT and non-robotic therapy on results of ADL at the end-of-treatment. The results showed that RAT could better improve the activity function at the end-of-treatment than controls (SMD = 0.32, 95% CI 0.16 to 0.47, P < 0.0001).

**Additional file 7: Fig S7.** Comparison of the effect of RAT and non-robotic therapy on results of ADL at the follow-up (≥ 3 months). The results indicated that long-term effect of RAT on ADL was similar with controls (SMD = 0.09, 95% CI -0.06 to 0.23, P = 0.25).

**Additional file 8: Fig S8.** The funnel plots of the results of the FM-UE and ADL at the end-of-treatment and at the follow-up. (A). The funnel plot of the outcomes of the FM-UE at the end-of-treatment;(B). The funnel plot of the outcomes of the FM-UE at the follow-up;(C). The funnel plot of the outcome of the ADL at the end-of-treatment; (D). The funnel plot of the outcome of the ADL at the follow-up.

**Additional file 9: Fig S9.** The sensitivity analysis of the outcomes of the FM-UE at the end-of-treatment.

**Additional file 10: Fig S10.** The sensitivity analysis of the outcomes of the FM-UE at the follow-up.

**Additional file 11: Fig S11.** The sensitivity analysis of the outcomes of ADL at the end-of-treatment.

**Additional file 12: Fig S12.** The sensitivity analysis of the outcomes of the ADL at the follow-up.

**Additional** **file** **13**:** Fig** **S13**. The subgroup analysis of the effect of RAT versus non-robotic therapy on outcome of FM-UE at the end-of-treatment in patients with different level of impairment. The results indicated that RAT had the additional benefit on motor control in patients with mild-to moderate paralysis (SMD = 0.26, 95% CI 0.09 to 0.42, P = 0.002), and had no significant clinical benefits in patients with severe paralysis (SMD = 0.14, 95% CI -0.01 to 0.30, P = 0.08).

## Data Availability

Not applicable.

## Abbreviations

RAT: Robot-assisted therapy
FM-UE: Fugl-Meyer Upper Extremity
ICF: International Classification of Functioning, Disability, and Health
PNF: Proprioceptive neuromuscular facilitation
ADL: Activity of daily living
FIM: Functional Independence Measure
BI: Barthel Index score
mBI: Modified Barthel Index score
SIS: Stroke Impact Scale
FAT: Frenchay Arm Test
SMD: Standard mean difference
EMG: Electromyography
