# Supplementary figures and images for: Short and long-term effects of robot-assisted therapy on upper limb motor function and activity of daily living in patients post-stroke: a meta-analysis of randomized controlled trials
Source: J Neuroeng Rehabil. 2022 Jul 21;19:76. doi: 10.1186/s12984-022-01058-8 (PMC9306153; doi:10.1186/s12984-022-01058-8)

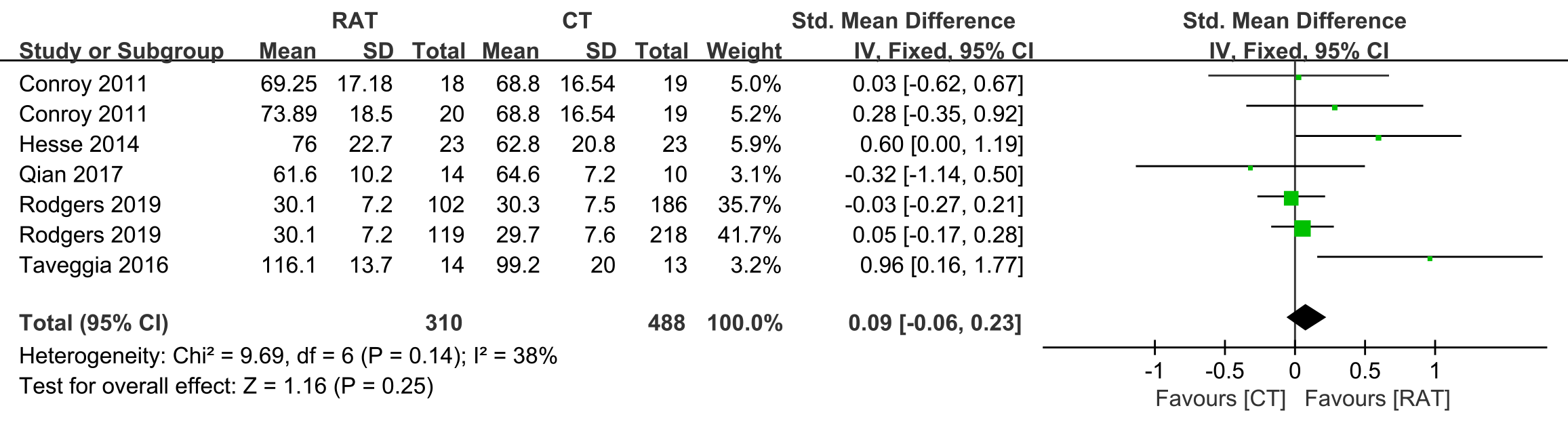

Supplement: Supplementary file 1 — Additional file 1: Fig S1. Flow diagram of study selection. [file 12984_2022_1058_MOESM1_ESM.tif]

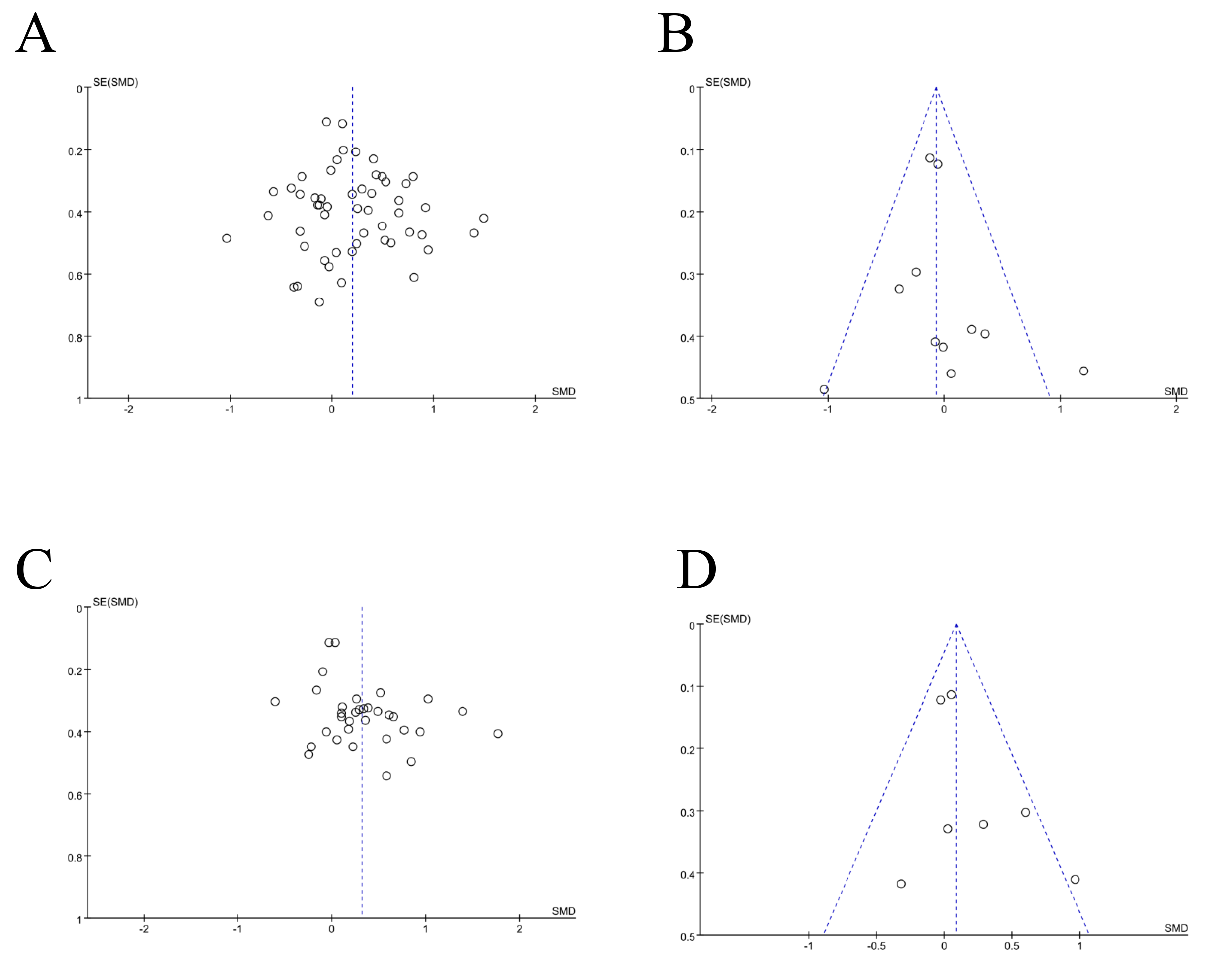

Supplement: Supplementary file 2 — Additional file 2: Fig S2. Risk of bias summary for all included studies. [file 12984_2022_1058_MOESM2_ESM.tif]

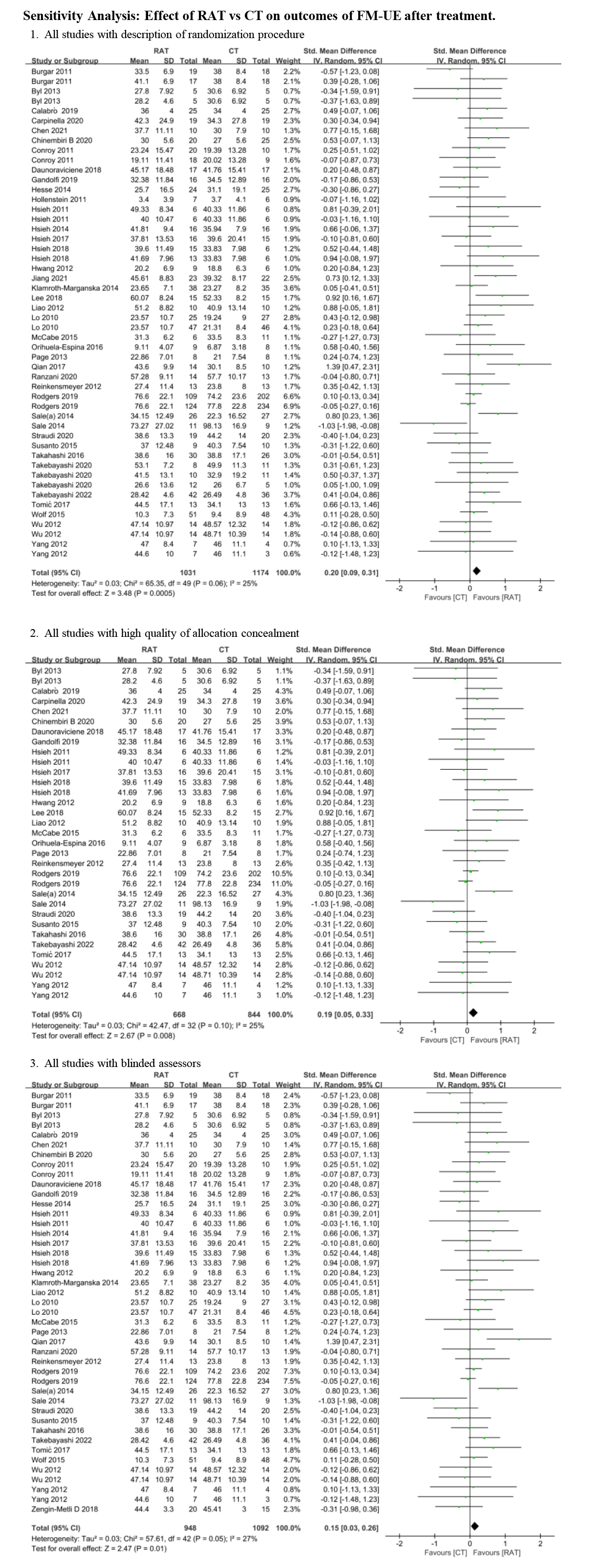

Supplement: Supplementary file 3 — Additional file 3: Fig S3. Risk of bias graph for all included studies. [file 12984_2022_1058_MOESM3_ESM.tif]

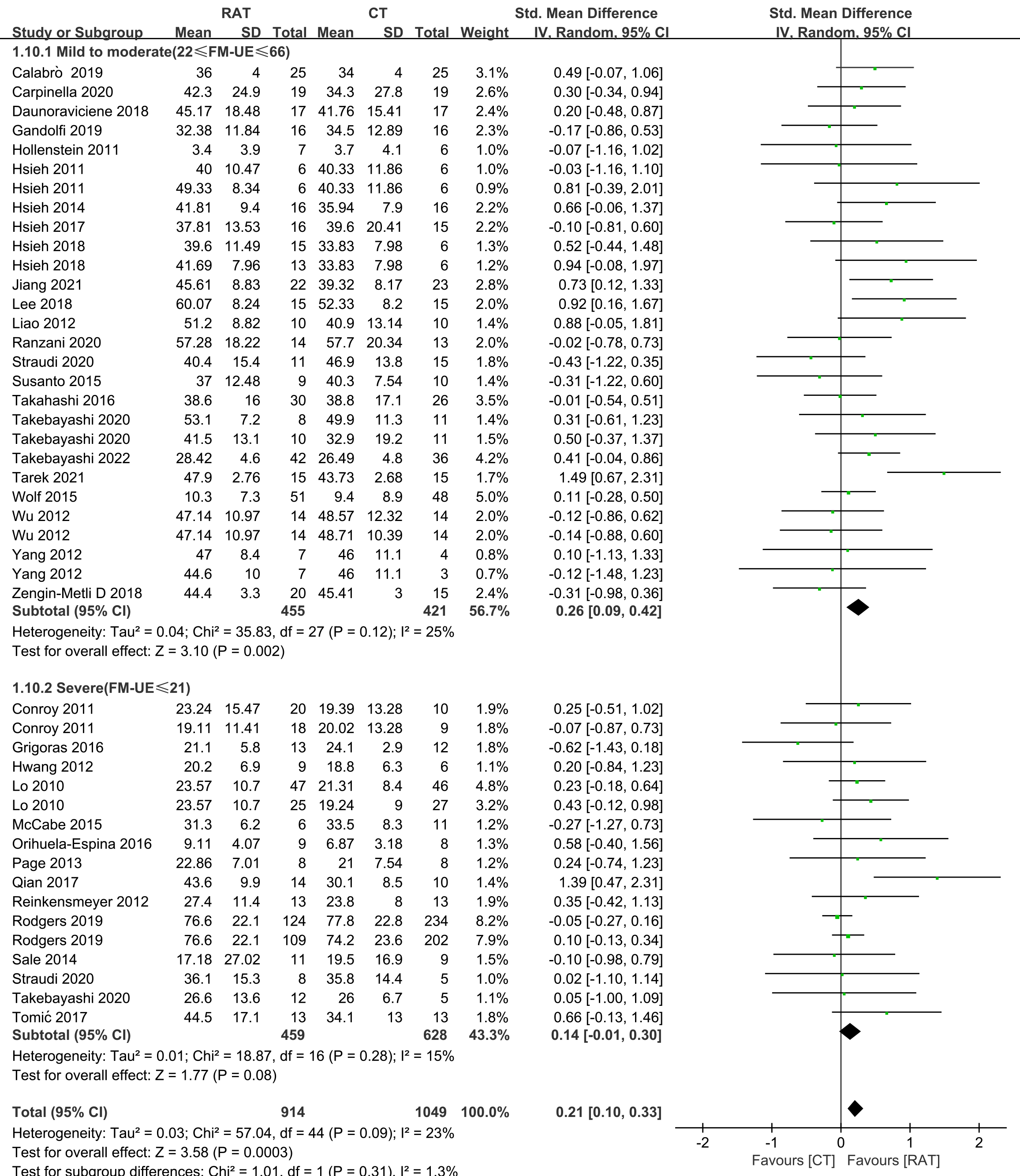

Supplement: Supplementary file 4 — Additional file 4: Fig S4. Comparison of the effect of RAT and non-robotic therapy on outcome of FM-UE scale at the end-of-treatment. The result showed that RAT had the additional immediated benfits on motor control compared with controls (SMD = 0.20, 95% CI 0.08 to 0.32, P = 0.001). [file 12984_2022_1058_MOESM4_ESM.tif]

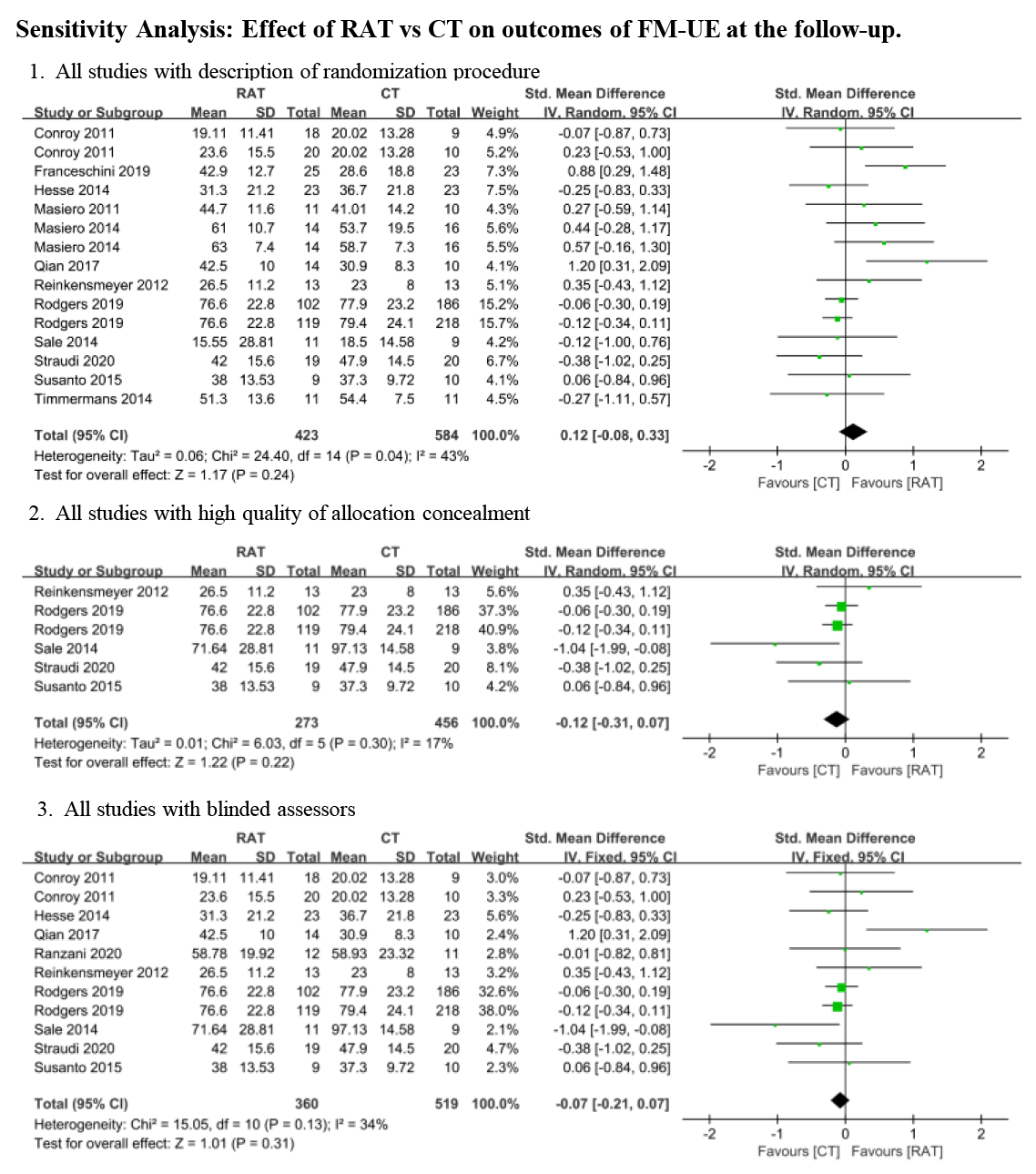

Supplement: Supplementary file 5 — Additional file 5: Fig 5. Comparison of the effect of RAT and non-robotic therapy on outcome of FM-UE at the follow-up (≥ 3 months). The result showed that the long-term effect of RAT on motor control was same as controls (SMD = -0.07, 95% CI -0.21 to 0.07, P = 0.31). [file 12984_2022_1058_MOESM5_ESM.tif]

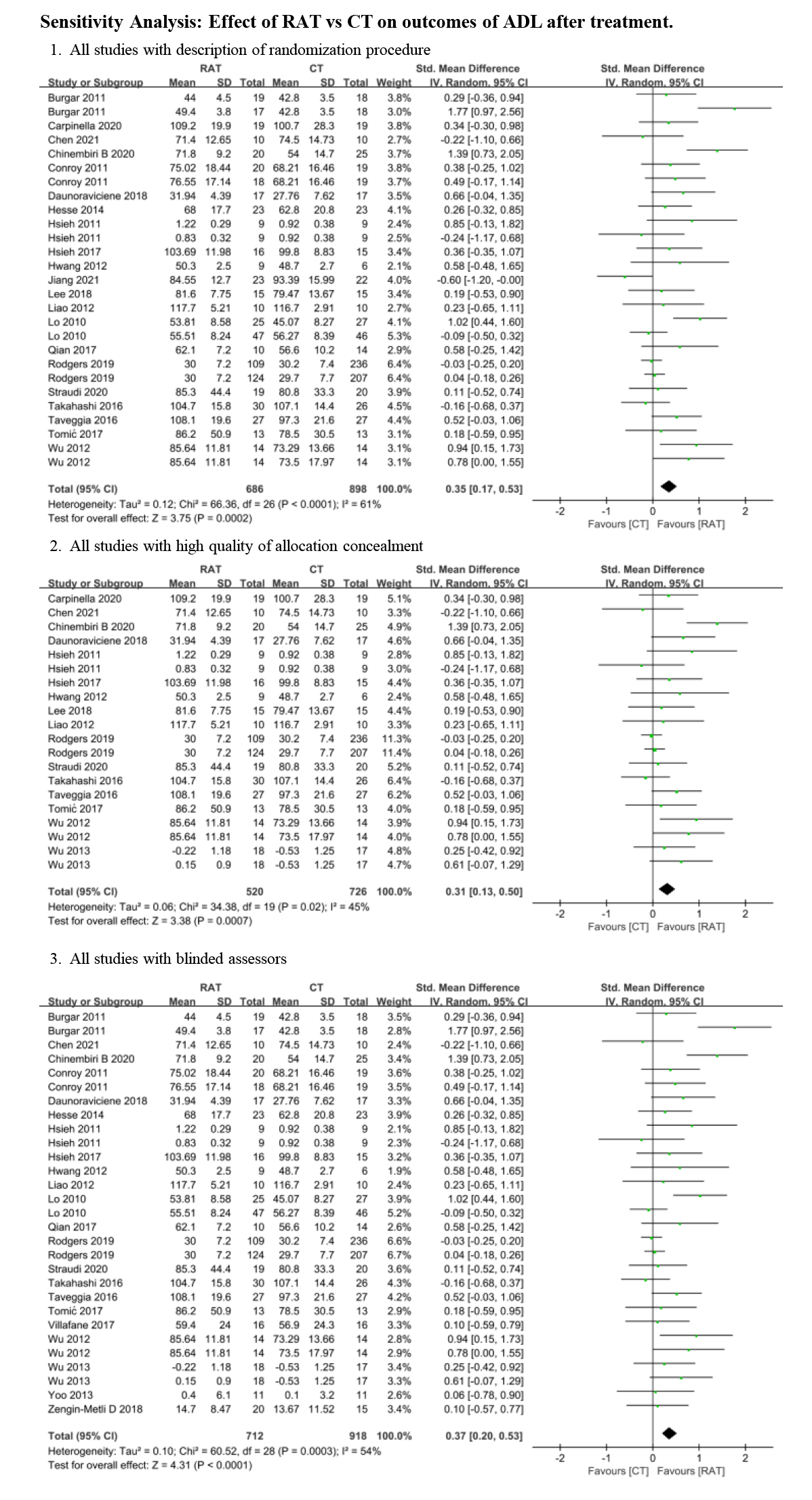

Supplement: Supplementary file 6 — Additional file 6: Fig S6. Comparison of the effect of RAT and non-robotic therapy on results of ADL at the end-of-treatment. The results showed that RAT could better improve the activity function at the end-of-treatment than controls (SMD = 0.32, 95% CI 0.16 to 0.47, P < 0.0001). [file 12984_2022_1058_MOESM6_ESM.tif]

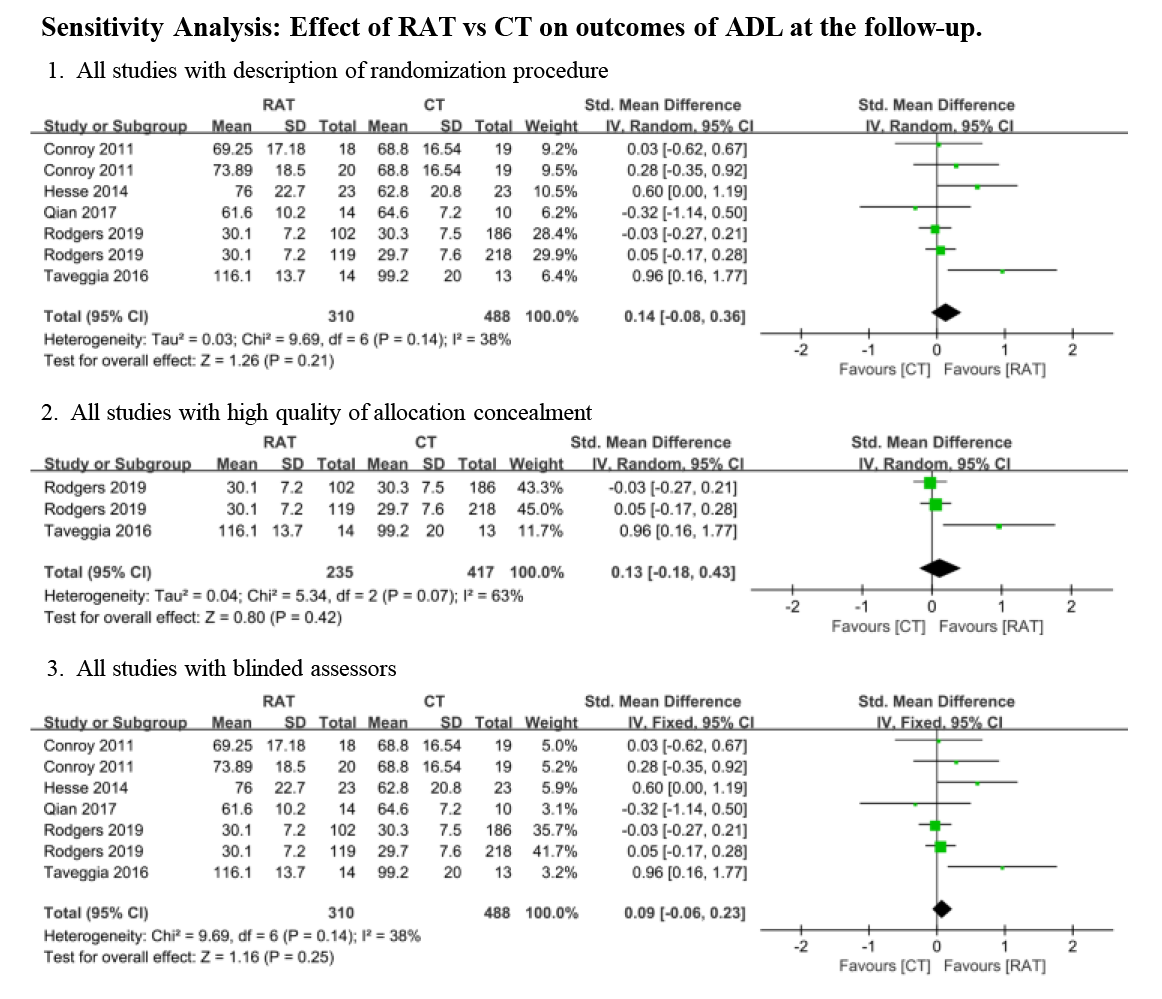

Supplement: Supplementary file 7 — Additional file 7: Fig S7. Comparison of the effect of RAT and non-robotic therapy on results of ADL at the follow-up (≥ 3 months). The results indicated that long-term effect of RAT on ADL was similar with controls (SMD = 0.09, 95% CI -0.06 to 0.23, P = 0.25). [file 12984_2022_1058_MOESM7_ESM.tif]

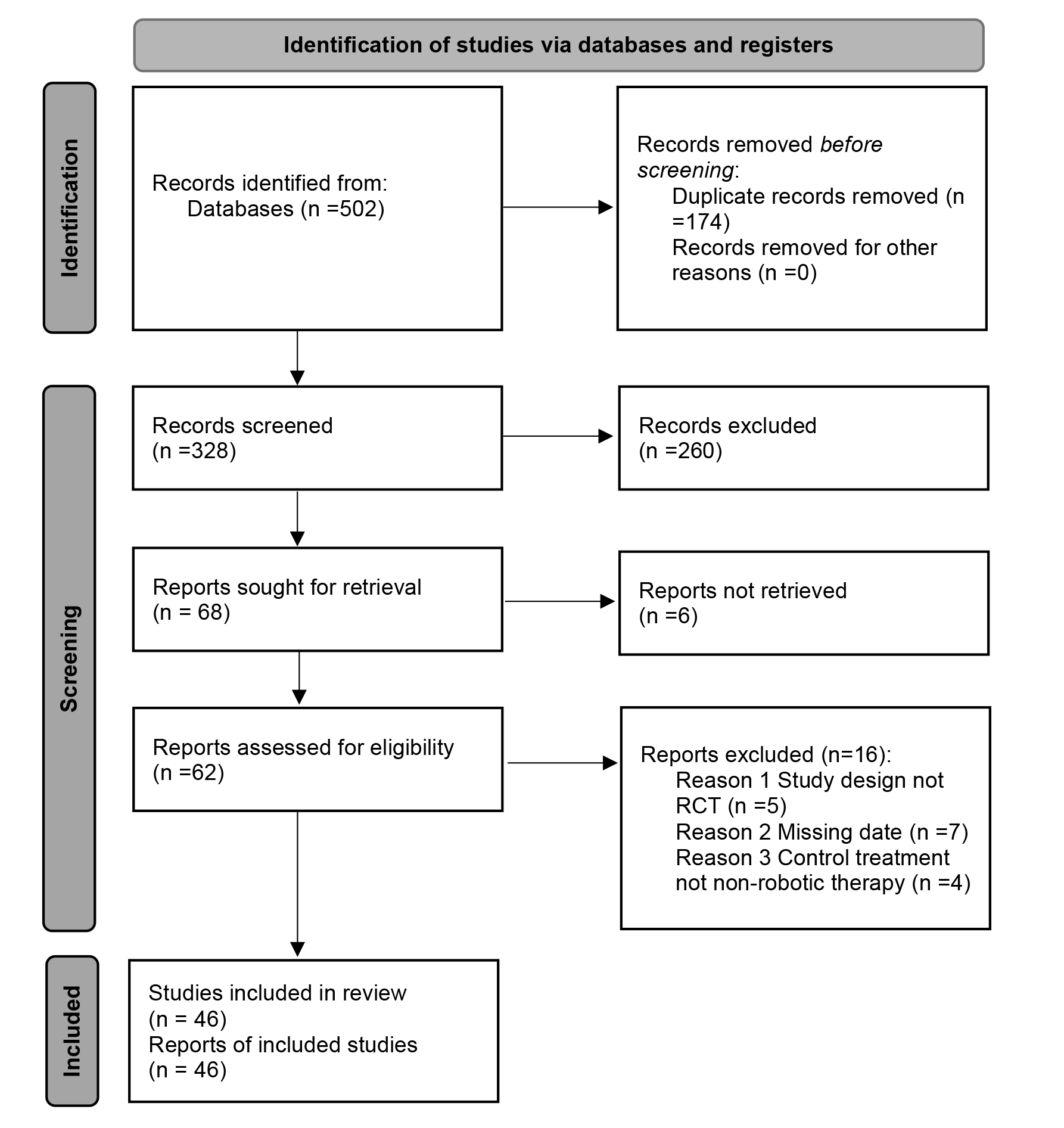

Supplement: Supplementary file 8 — Additional file 8: Fig S8. The funnel plots of the results of the FM-UE and ADL at the end-of-treatment and at the follow-up. (A). The funnel plot of the outcomes of the FM-UE at the end-of-treatment;(B). The funnel plot of the outcomes of the FM-UE at the follow-up;(C). The funnel plot of the outcome of the ADL at the end-of-treatment; (D). The funnel plot of the outcome of the ADL at the follow-up. [file 12984_2022_1058_MOESM8_ESM.tif]

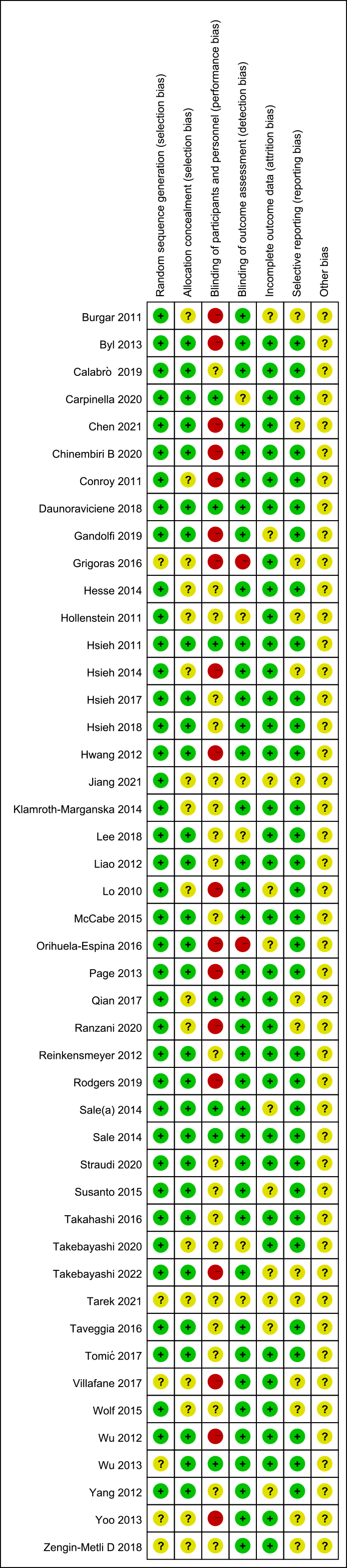

Supplement: Supplementary file 9 — Additional file 9: Fig S9. The sensitivity analysis of the outcomes of the FM-UE at the end-of-treatment. [file 12984_2022_1058_MOESM9_ESM.tif]

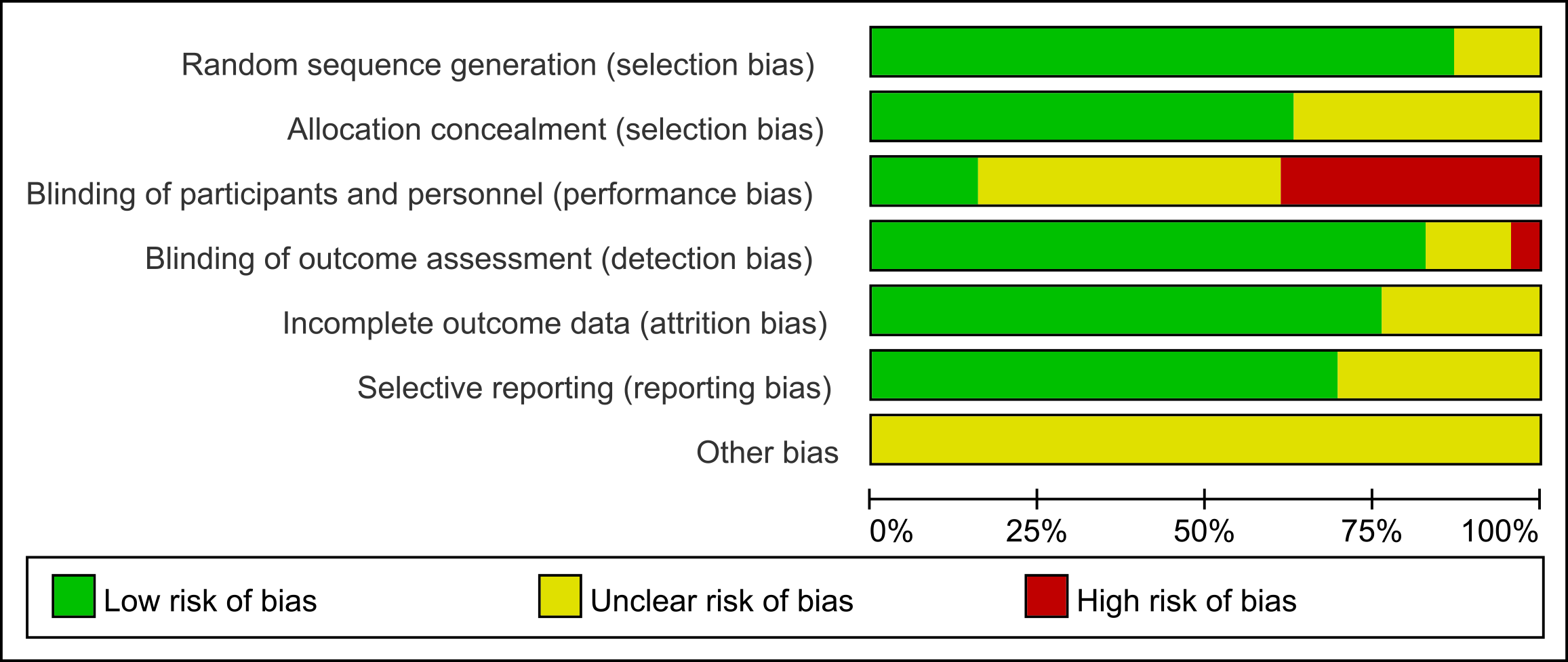

Supplement: Supplementary file 10 — Additional file 10: Fig S10. The sensitivity analysis of the outcomes of the FM-UE at the follow-up. [file 12984_2022_1058_MOESM10_ESM.tif]

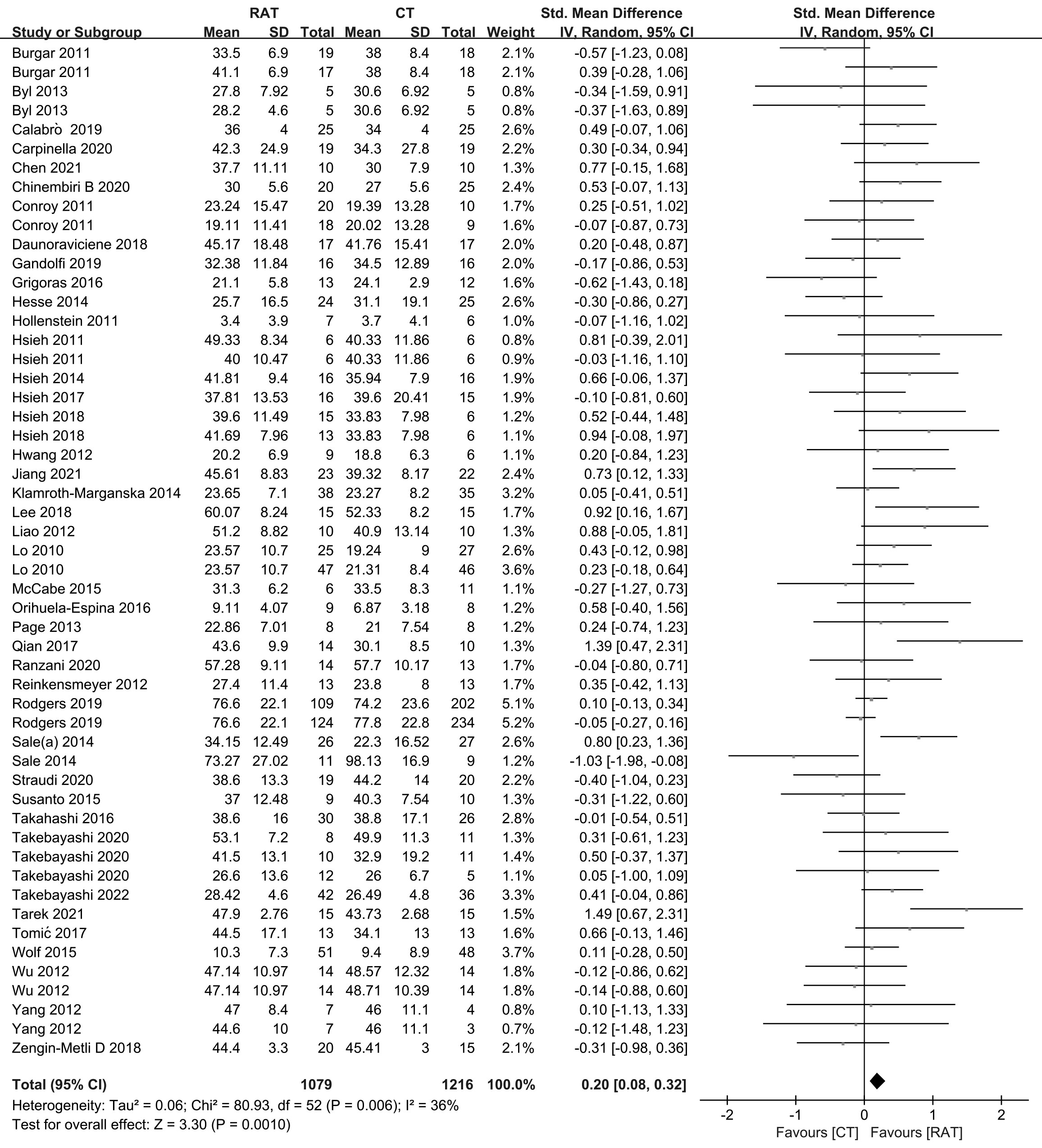

Supplement: Supplementary file 11 — Additional file 11: Fig S11. The sensitivity analysis of the outcomes of ADL at the end-of-treatment. [file 12984_2022_1058_MOESM11_ESM.tif]

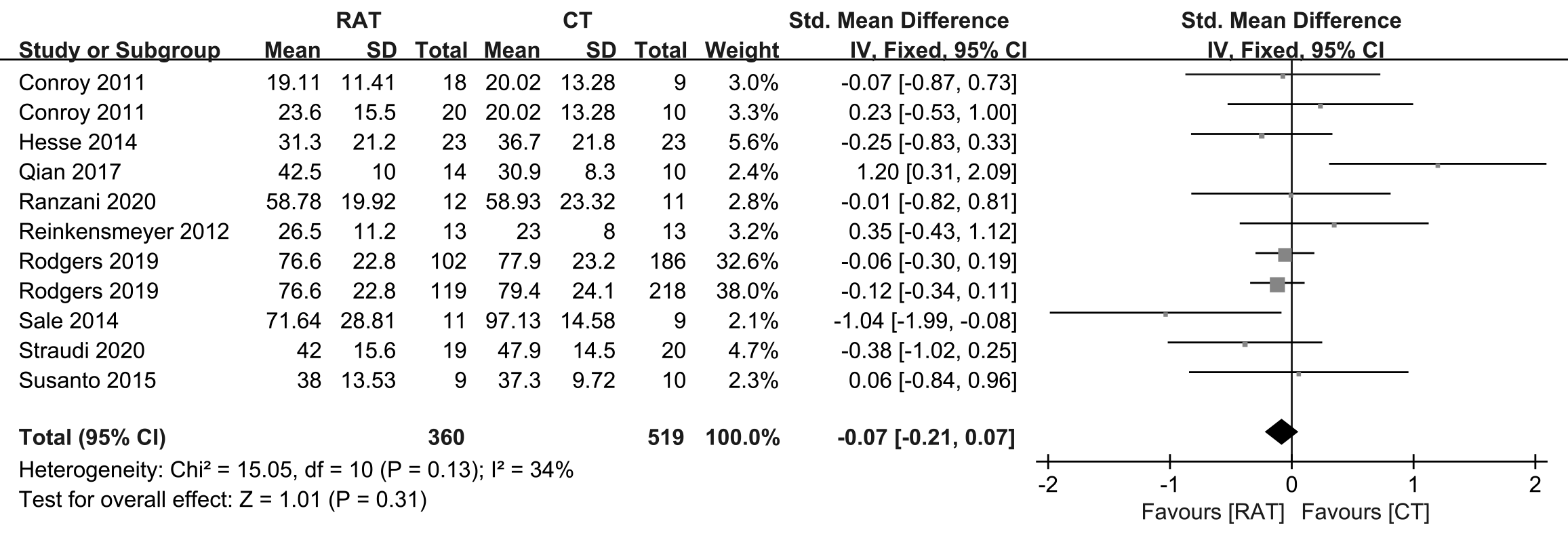

Supplement: Supplementary file 12 — Additional file 12: Fig S12. The sensitivity analysis of the outcomes of the ADL at the follow-up. [file 12984_2022_1058_MOESM12_ESM.tif]

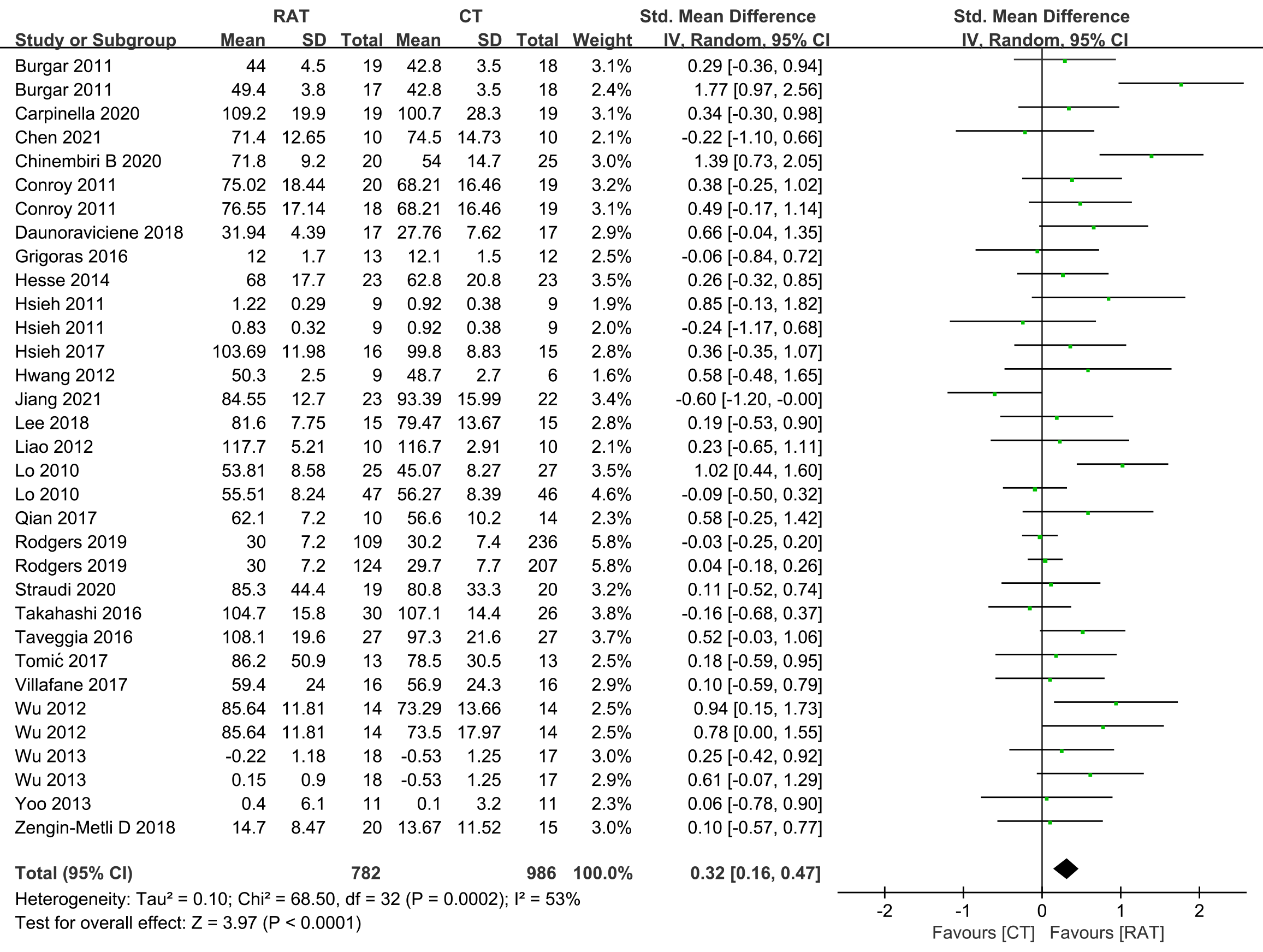

Supplement: Supplementary file 13 — Additional file 13: Fig S13. The subgroup analysis of the effect of RAT versus non-robotic therapy on outcome of FM-UE at the end-of-treatment in patients with different level of impairment. The results indicated that RAT had the additional benefit on motor control in patients with mild-to moderate paralysis (SMD = 0.26, 95% CI 0.09 to 0.42, P = 0.002), and had no significant clinical benefits in patients with severe paralysis (SMD = 0.14, 95% CI -0.01 to 0.30, P = 0.08). [file 12984_2022_1058_MOESM13_ESM.tif]
